# The function of lactate dehydrogenase A in retinal neurons: implications to retinal degenerative diseases

**DOI:** 10.1093/pnasnexus/pgad038

**Published:** 2023-02-03

**Authors:** Ammaji Rajala, Mohd A Bhat, Kenneth Teel, Gopa Kumar Gopinadhan Nair, Lindsey Purcell, Raju V S Rajala

**Affiliations:** Department of Ophthalmology, University of Oklahoma Health Sciences Center, 608 Stanton L. Young Blvd, Oklahoma City, OK 73104, USA; Dean McGee Eye Institute, 608 Stanton L. Young Blvd, Oklahoma City, OK 73104, USA; Department of Ophthalmology, University of Oklahoma Health Sciences Center, 608 Stanton L. Young Blvd, Oklahoma City, OK 73104, USA; Dean McGee Eye Institute, 608 Stanton L. Young Blvd, Oklahoma City, OK 73104, USA; Department of Ophthalmology, University of Oklahoma Health Sciences Center, 608 Stanton L. Young Blvd, Oklahoma City, OK 73104, USA; Dean McGee Eye Institute, 608 Stanton L. Young Blvd, Oklahoma City, OK 73104, USA; Department of Ophthalmology, University of Oklahoma Health Sciences Center, 608 Stanton L. Young Blvd, Oklahoma City, OK 73104, USA; Dean McGee Eye Institute, 608 Stanton L. Young Blvd, Oklahoma City, OK 73104, USA; Department of Ophthalmology, University of Oklahoma Health Sciences Center, 608 Stanton L. Young Blvd, Oklahoma City, OK 73104, USA; Dean McGee Eye Institute, 608 Stanton L. Young Blvd, Oklahoma City, OK 73104, USA; Department of Ophthalmology, University of Oklahoma Health Sciences Center, 608 Stanton L. Young Blvd, Oklahoma City, OK 73104, USA; Dean McGee Eye Institute, 608 Stanton L. Young Blvd, Oklahoma City, OK 73104, USA; Department of Physiology, University of Oklahoma Health Sciences Center, 608 Stanton L. Young Blvd, Oklahoma City, OK 73104, USA; Department of Cell Biology, University of Oklahoma Health Sciences Center, 608 Stanton L. Young Blvd, Oklahoma City, OK 73104, USA

**Keywords:** aerobic glycolysis, retina, photoreceptors, lactate dehydrogenases, Warburg effect

## Abstract

The postmitotic retina is highly metabolic and the photoreceptors depend on aerobic glycolysis for an energy source and cellular anabolic activities. Lactate dehydrogenase A (LDHA) is a key enzyme in aerobic glycolysis, which converts pyruvate to lactate. Here we show that cell-type-specific actively translating mRNA purification by translating ribosome affinity purification shows a predominant expression of LDHA in rods and cones and LDHB in the retinal pigment epithelium and Müller cells. We show that genetic ablation of LDHA in the retina resulted in diminished visual function, loss of structure, and a loss of dorsal–ventral patterning of the cone-opsin gradient. Loss of LDHA in the retina resulted in increased glucose availability, promoted oxidative phosphorylation, and upregulated the expression of glutamine synthetase (GS), a neuron survival factor. However, lacking LDHA in Müller cells does not affect visual function in mice. Glucose shortage is associated with retinal diseases, such as age-related macular degeneration (AMD), and regulating the levels of LDHA may have therapeutic relevance. These data demonstrate the unique and unexplored roles of LDHA in the maintenance of a healthy retina.

**Significance Statement**

Lactate dehydrogenase (LDH) is a critical enzyme during aerobic glycolysis. Here we investigate which LDH isoforms are expressed in retinal cells and whether ablating LDHA in the retina can disrupt photoreceptor functions. We also show predominant expression of LDHA in rods and cones and LDHB in Müller cells and retinal pigment epithelial cells. We show that mice lacking LDHA, while normal at a young age, exhibit age-related decline of function and structural defects. Loss of LDHA in the retina resulted in increased glucose availability and promoted oxidative phosphorylation, altered the cone-opsin gradient, and upregulated the expression of GS. Glucose shortage is associated with retinal diseases, such as AMD. Regulating the levels of LDHA may have therapeutic relevance.

## Introduction

The postmitotic retina remains metabolically active and is comparable to rapidly proliferating mitotic tumor cells (1, 2). Aerobic glycolysis is potentially important for providing energy supply and cellular anabolism to photoreceptor cells (3–7). Similar to cancer cells, photoreceptor cells produce lactate aerobically through lactate dehydrogenase A (LDHA), which converts pyruvate to lactate and utilizes NADH as a cofactor (8, 9). The lactate can be converted to pyruvate by the action of lactate dehydrogenase B (LDHB), which utilizes NAD as a cofactor (10). Earlier studies show that LDHA is expressed in primate and rodent photoreceptor cells (11). In developing chick retina, LDHB is shown to localize to aerobic regions of the retina where active mitochondria are located (12). LDHA is a hypoxia-inducible gene, whereas LDHB expression is repressed by hypoxic conditions in primary retina cultures of rats and chicks (12). The localization of LDHA in the context of LDHB and *vice versa* is not known. A previous study showed that lactate production in the photoreceptors occurs in an LDHA-dependent manner (3). Furthermore, the functional role of LDHA in the retina and photoreceptors is not established. However, rod-specific deletion of basigin, an accessory protein associated with monocarboxylate transporters (MCT 1, 3, and 4) that transport lactate, resulted in retinal degeneration (9).

In the present study, we investigated the localization and developmental expression of LDHA and LDHB and found that LDHA expression is localized to the outer retina, whereas LDHB expression is localized to the inner retina. Affinity purification of translated mRNAs from the rods, cones, Müller cells, and retinal pigment epithelium (RPE) using RiboTag mice showed that LDHA is predominantly expressed in rods and cones compared with Müller cells and RPE, whereas RPE and Müller cells express significantly higher levels of LDHB than do rods and cones. Retina-specific deletion of LDHA showed no effect on the retina structure, but produced a significant loss of rod function in 28-week-old mice. However, at 40 weeks of age, LDHA knockout mice exhibit photoreceptor degeneration. We found increased pyruvate kinase activity and increased ATP levels in LDHA knockout mice due to the downregulation of Warburg mediator PKM2, which resulted in the upregulation of PKM1. We found significantly increased expression of glutamine synthetase (GS), which correlated with increased glutamine levels in LDHA knockout retinas. Our studies also showed that LDHA regulates the dorsal–ventral cone-opsin gradient. Furthermore, Müller cell-specific deletion of LDHA did not affect retina function. These studies for the first time demonstrate the unique roles of LDHA in the maintenance of a healthy retina.

## Results

### Developmentally regulated expression of LDHA and LDHB isoforms

**Fig. 1.**
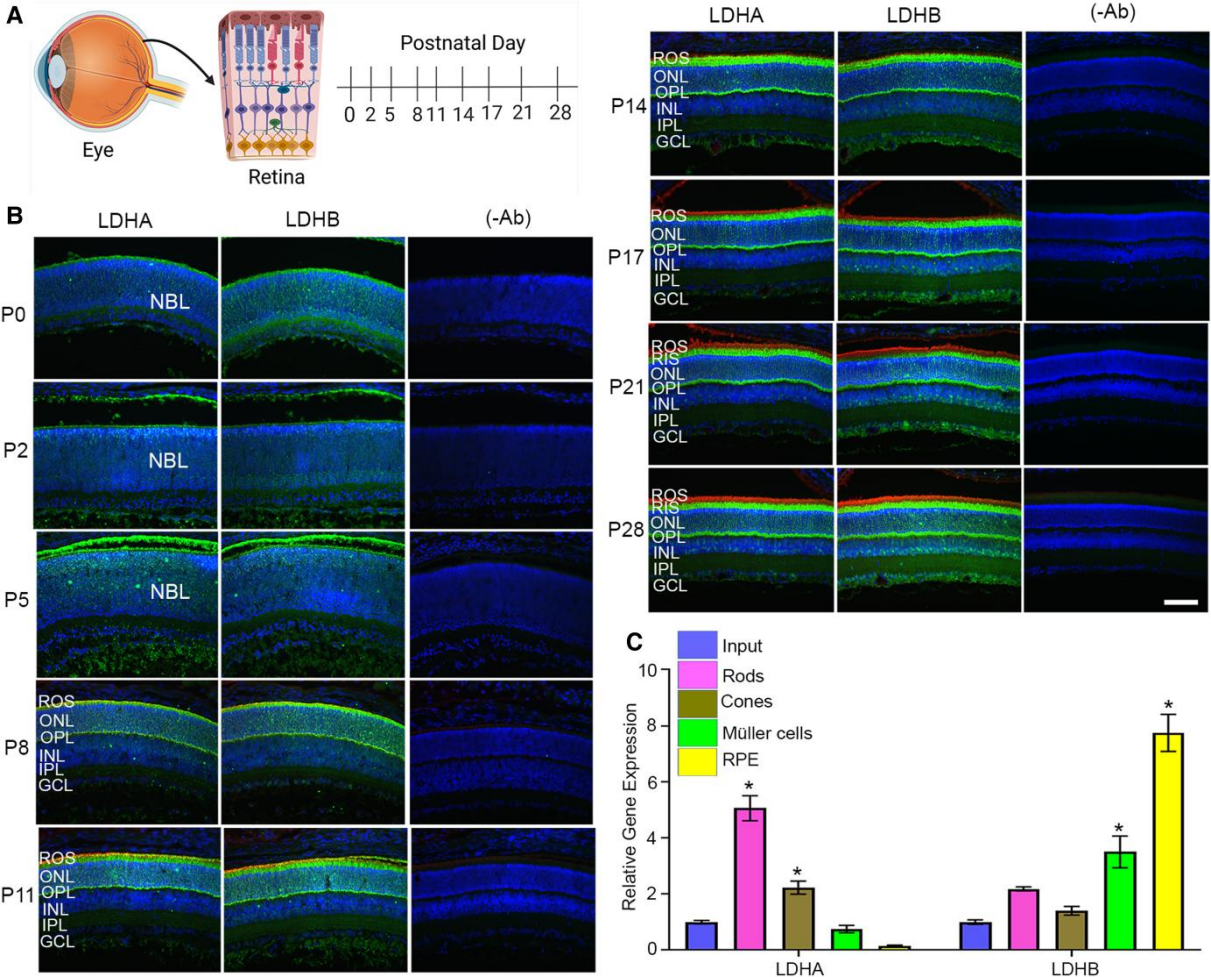
Developmental expression of LDHA and LDHB in the retina. Cartoon showing the cross-section of the eye and time points where the mouse eyes were harvested and fixed (A). Mouse retinal sections prepared at postnatal day (P) 0, P2, P5, P8, P11, P14, P17, P21, and P28 were stained with LDHA (left panel), LDHB (middle panel) and rhodopsin antibodies (B). Omission of primary antibodies (-Ab, right panel). NBL, neuroblastic layer; ROS, rod outer segments; ONL, outer nuclear layer; OPL, outer plexiform layer; INL, inner nuclear layer; IPL, inner plexiform layer; GCL, ganglion cell layer. Scale bar = 50 μm. Equal amounts of mRNA from the retina, rod photoreceptor, cone photoreceptor, Müller, and RPE cells were used to measure the quantitative expression of *Ldha* and *Ldhb* by qRT-PCR and we normalized the expression to *Rpl38* and *β-actin* levels (C). The mRNA levels were averaged and data were expressed as mean ± *SEM (n* = 3). Panels A was created with BioRender.com. *P < 0.001.

To investigate the expression of LDHA and LDHB during postnatal development, mouse retinal sections were prepared at postnatal day 0 (P0), P2, P5, P8, P11, P14, P17, P21, and P28 and were immunostained for LDHA and LDHB (Fig. 1A). At P0, LDHA expression was observed in the neuroblastic layer (NBL), whereas LDHB expression was absent (Fig. 1B). The expression of LDHA, but not LDHB, increased at P2 and P5 (Fig. 1B). At P8, LDHA expression was observed in the photoreceptor layer, and ganglion cells showed the expression of LDHB (Fig. 1B). Rhodopsin expression began to appear around P5. By P11, rod outer segments (ROS) showed a predominant expression of rhodopsin. At P11, LDHA expression was noted in the outer segments, inner segments, and outer nuclear layer (ONL), whereas LDHB expression was observed in the inner plexiform and ganglion cell layers (Fig. 1B). This trend continued up to P14 (Fig. 1B). At P17, LDHA expression was predominantly localized to rod inner segments (RIS) and the outer nuclear and outer plexiform layers, whereas LDHB expression was predominantly observed in the inner nuclear, inner plexiform, and ganglion cell layers (Fig. 1B). The expression of LDHA and LDHB was similar to P17 at P21 and P28 (Fig. 1B). These observations suggest that LDHA expression starts earlier than LDHB during development, suggesting that LDHA is needed for aerobic glycolysis for macromolecule synthesis.

Actively translating mRNAs from rods, cones, Müller cells, and RPE showed that rods and cones predominantly express LDHA compared with Müller cells and RPE (Fig. 1C), whereas RPE and Müller cells predominantly express LDHB compared with rods and cones (Fig. 1C). These results suggest that in photoreceptor cells, pyruvate is converted to lactate, whereas in RPE and Müller cells, lactate is converted to pyruvate.

### Generation of retina-specific LDHA knockout mice and characterization of LDH isoforms in the retina

**Fig. 2.**
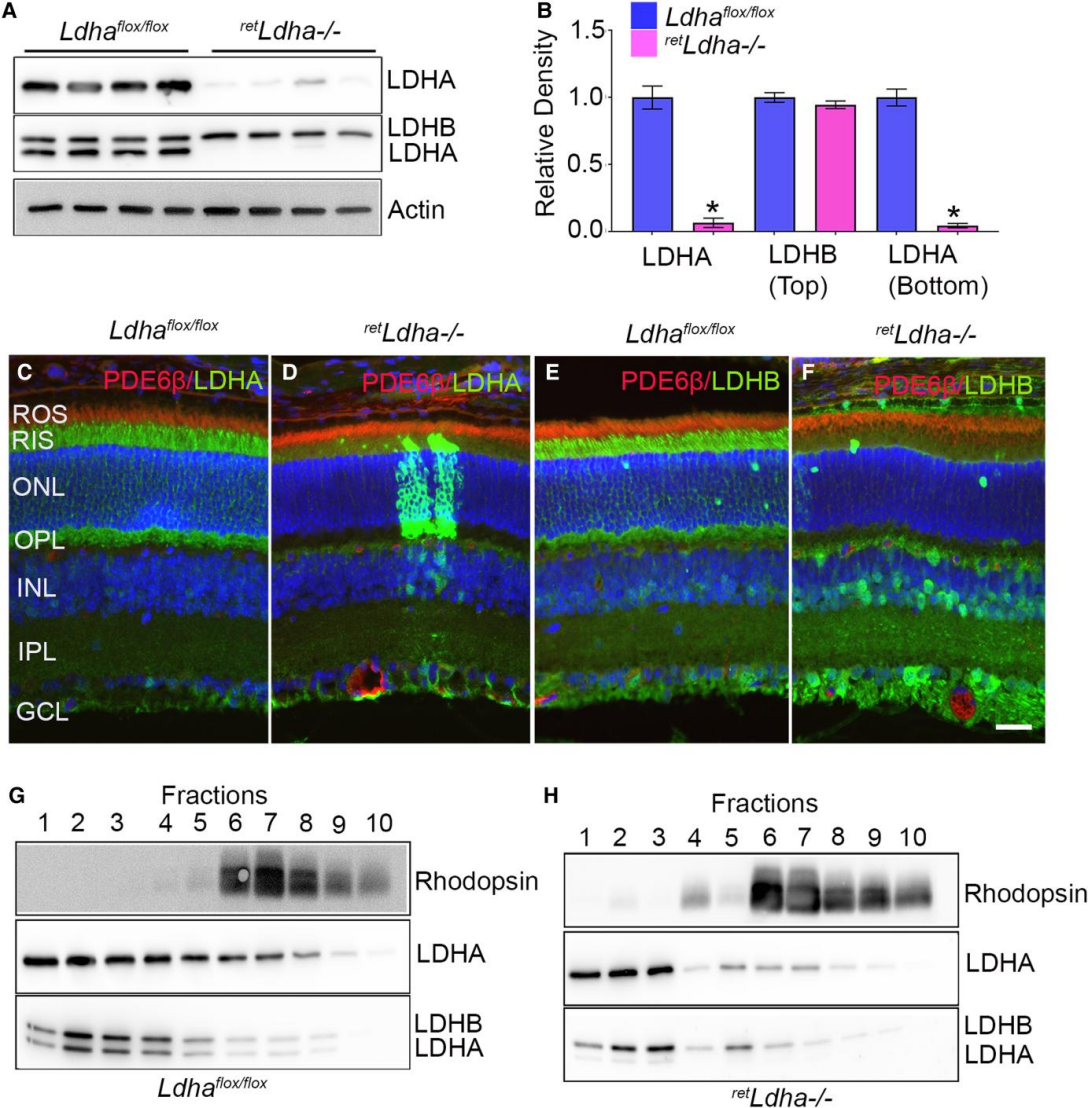
Characterization of LDHA and LDHB isoforms in the retina. Immunoblot analysis of 4-week-old *Ldha^flox/flox^* and *^ret^Ldha^−/−^* mouse retinas with LDHA, LDHB, and actin antibodies (A). Densitometric analysis of LDHA and LDHB were normalized to actin (B). Data are mean ± *SEM* (*n = 4*). *P < 0.0001. *Ldha^flox/flox^* (C, E) and *^ret^Ldha^−/−^* (D, F) mouse retina sections were stained for LDHA (C, D), LDHB (E, F), and PDE6β (C–F) antibodies. ROS, rod outer segments; RIS, rod inner segments; ONL, outer nuclear layer; OPL, outer plexiform layer; INL, inner nuclear layer; IPL, inner plexiform layer; GCL, ganglion cell layer. Scale bar = 50 μm. Retinal homogenates from *Ldha^flox/flox^* (G) and *^ret^Ldha^−/−^* (H) mouse retinas were subjected to OptiPrep (8–40%) density gradient centrifugation. Fractions of inner segments and intact photoreceptors were collected from the top to the bottom of the gradients. A 10 μL sample (1 μL for rhodopsin) was subjected to immunoblot analysis with rhodopsin, LDHA, and LDHB antibodies.

To investigate the role of LDHA in the retina, we generated retina-specific deletion of LDHA under the control of the Chx10 promoter (13). Chx10 is expressed at high levels in uncommitted retinal progenitor cells and mature bipolar cells (14). We used two LDH antibodies, one specific to LDHA (LDHA antibody) and the other recognizing both LDHA and LDHB isoforms (LDHB antibody). Immunoblot analysis of LDHA floxed (*Ldha^flox/flox^*) and knockout (*^ret^Ldha^−/−^*) mouse retinas showed that LDHA antibody recognized only the LDHA in *Ldha^flox/flox^* and LDHA expression is absent from the *^ret^Ldha^−/−^* mice (Fig. 2A, B). The LDHB antibody recognizes both LDHA (lower band) and LDHB (upper band); this antibody recognized LDHB in both *Ldha^flox/flox^* and *^ret^Ldha^−/−^* mice (Fig. 2A, B). LDHA is predominantly expressed in RIS and the outer plexiform layer (OPL) in *Ldha^flox/flox^* mice and is absent from *^ret^Ldha^−/−^* mice (Fig. 2C–D). LDHB antibody recognizes both LDHA and LDHB in *Ldha^flox/flox^* (Fig. 2E) mice. In *^ret^Ldha^−/−^* mice, LDHB is expressed below the OPL, inner nuclear layer, inner plexiform layer, and ganglion cell layer (Fig. 2F). Collectively, our studies suggest that LDHA is predominantly localized to the outer retina (Fig. 2C), whereas LDHB is predominantly localized to the inner retina (Fig. 2F). To further confirm the localization and loss of LDHA in photoreceptor cells, OptiPrep density gradient (8–40%) centrifugation was employed which yields ROS and broken inner segments (which release soluble cytoplasmic proteins) (15). Fractions 6–8 represent intact ROS/RIS fractions. The fractionation profile indicated a reduced protein level of LDHA in *^ret^Ldha^−/−^* mice (Fractions 4–10) (Fig. 2H) compared with *Ldha^flox/flox^* mice (Fig. 2G), suggesting that LDHA is localized to photoreceptors and further confirms the deletion of LDHA in *^ret^Ldha^−/^*mice. The presence of residual LDHA in *^ret^Ldha^−/−^* mouse photoreceptors (Fractions 1–3) could most likely come from other retinal cells or incomplete recombination of Cre-mediated excision of floxed LDHA allele. The majority of LDHB protein is migrated away from the rhodopsin, suggesting a non-photoreceptor origin (Fig. 2G, H).

### Structural and functional characterization of *^ret^Ldha^−/−^* mice

**Fig. 3.**
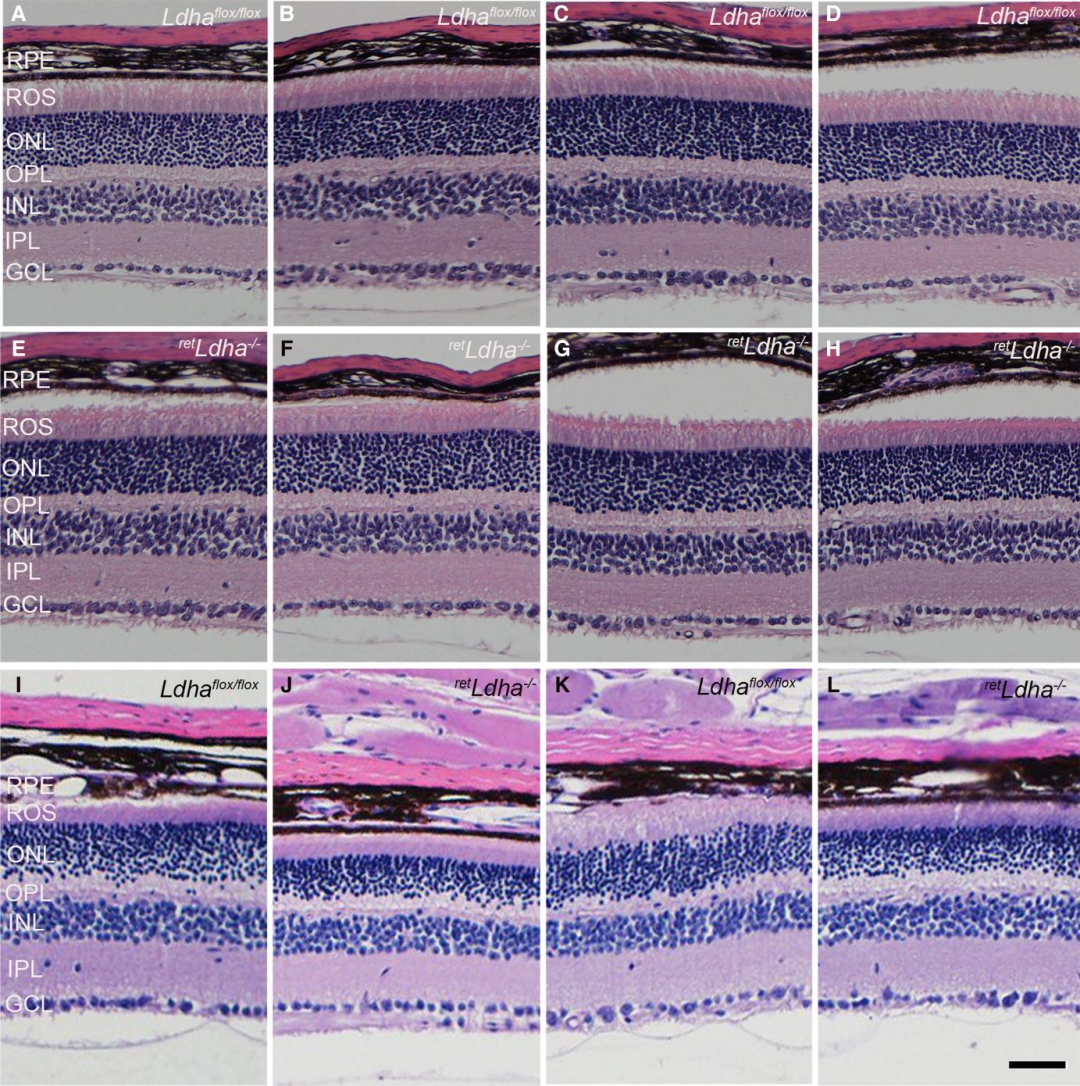
Structural characterization of *^ret^Ldha^−/−^* mouse retina. Four individual 4-week-old *Ldha^flox/flox^* (A–D) and *^ret^Ldha^−/−^* (E–H) and two individual 40-week-old *Ldha^flox/flox^* (I, K) and *^ret^Ldha^−/−^* (J, L) mouse retinas were stained with hematoxylin and eosin and examined for morphology. Scale bar = 50 μm.

**Fig. 4.**
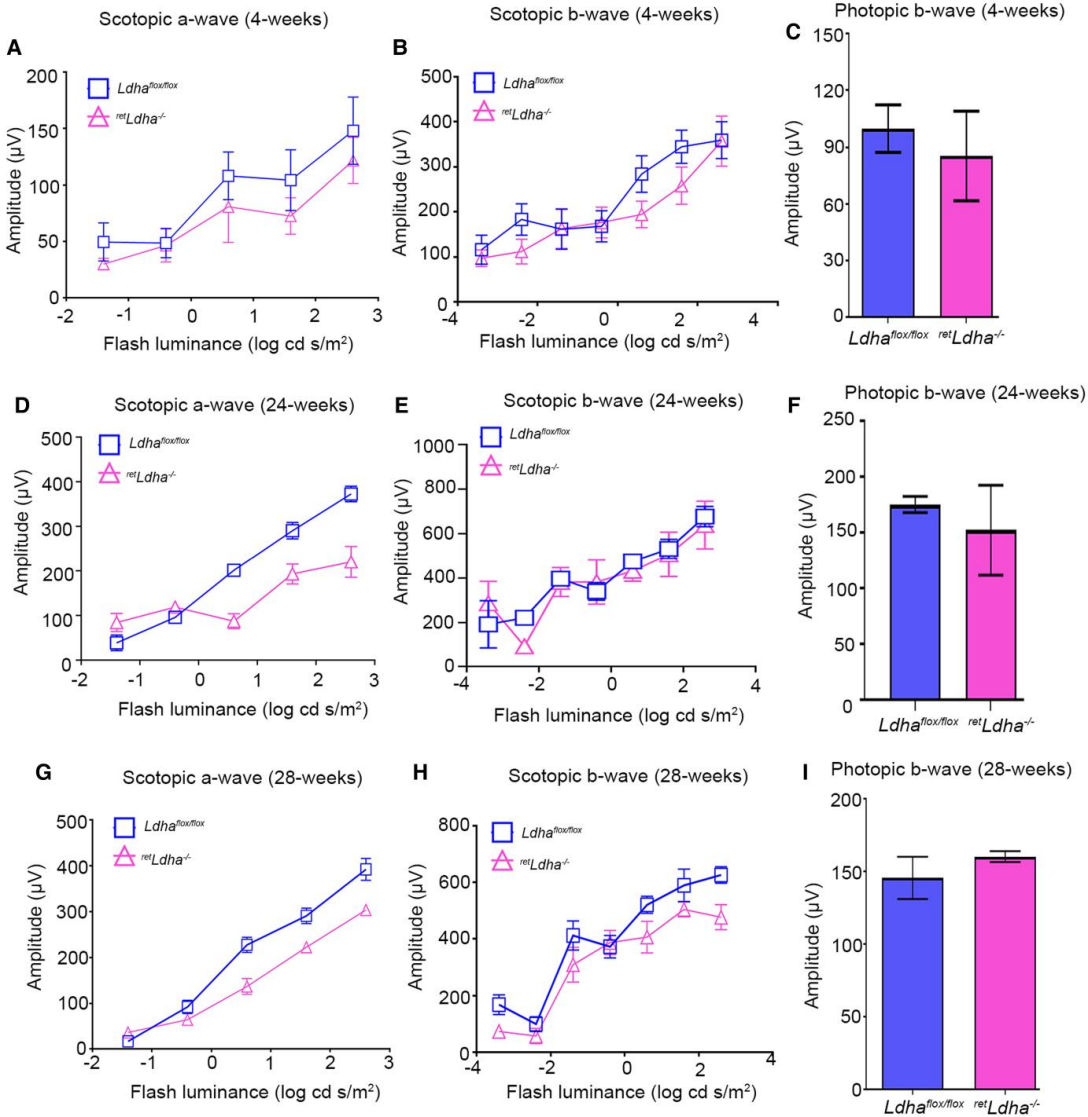
Functional characterization of *^ret^Ldha^−/−^* mouse retina. Scotopic a-wave (A, D, G), scotopic b-wave (B, E, H), and photopic b-wave (C, F, I) analyses were performed on 4-week-old (A–C), 24-week-old (D–F), and 28-week-old (G–I) *Ldha^flox/flox^* and *^ret^Ldha^−/−^* mice. Data are mean ± *SEM* (*n* = 8). The *^ret^Ldha^−/−^* response was significantly lower than that of the *Ldha^flox/flox^* retinas (*P* < 0.001). Data were analyzed by two-way ANOVA and unpaired *t*-test.

To determine the effect of loss of LDHA on retina structure, retina sections from 4-week-old *Ldha^flox/flox^* (Fig. 3A–D) and *^ret^Ldha^−/−^* mice (Fig. 3E–H) were stained with hematoxylin and eosin and we examined the morphology. The retina appeared normal between the two genotypes. There was no loss of ONL thickness, which is a measure of retinal degeneration, in *^ret^Ldha^−/−^* mice (16). The retinal cell integrity was well preserved (Fig. 3A–H). These observations suggest that retinal cell viability was not affected in *^ret^Ldha^−/−^* mice. In older mice, at 40 weeks of age, thinning of outer nuclear thickness was observed in *^ret^Ldha^−/−^* mice (Fig. 3J, L) but not in *Ldha*^flox/flox^ (Fig. 3I, K), suggesting photoreceptor degeneration resulting from loss of LDHA. At 4 weeks old, there was no significant difference in the scotopic a-wave and scotopic b-wave amplitudes, which represent rod function, and photopic b-wave amplitudes, which represents cone function in *^ret^Ldha^−/−^* mice, compared with *Ldha^flox/flox^* mice (Fig. 4A–C). Twenty-four-week-old *^ret^Ldha^−/−^* mice exhibit a significant loss of scotopic a-wave amplitude compared with *Ldha^flox/flox^* mice (Fig. 4D). However, there was no significant difference in scotopic b-wave and photopic b-wave amplitudes between *Ldha^flox/flox^* and *^ret^Ldha^−/−^* mice at this age (Fig. 4E, F). Twenty-eight-week-old *^ret^Ldha^−/−^* mice exhibited a significant loss of both scotopic a-and b-wave amplitudes compared with *Ldha^flox/flox^* mice (Fig. 4G, H). However, there was no significant difference in photopic b-wave amplitudes between *Ldha^flox/flox^* and *^ret^Ldha^−/−^* mice (Fig. 4I).

### Effect of loss of LDHA on photoreceptor and Müller cell-protein expression

**Fig. 5.**
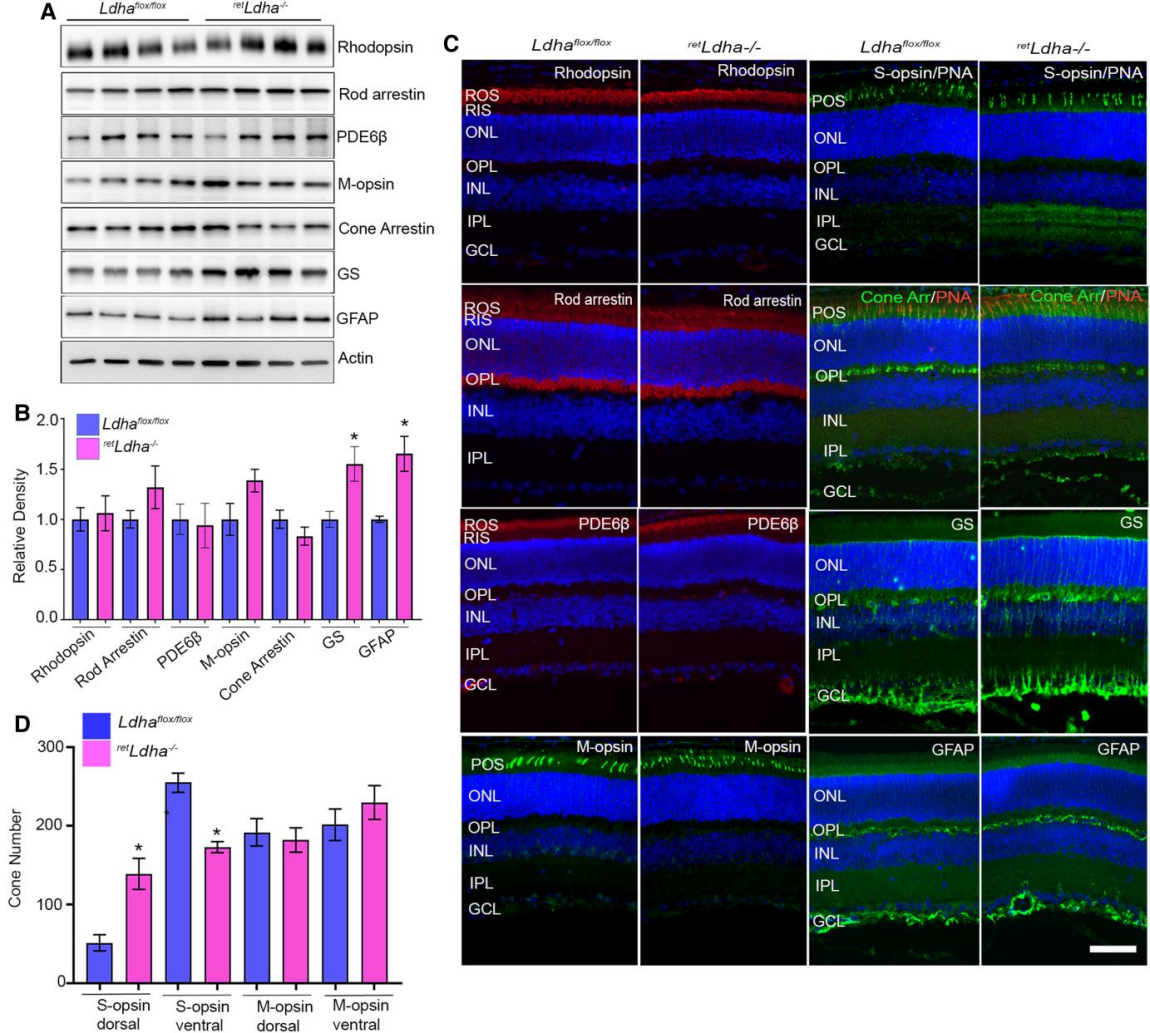
Effect of loss of LDHA on photoreceptor and Müller cell-protein expression. Immunoblot analysis of 4-week-old *Ldha^flox/flox^* and *^ret^Ldha^−/−^* mouse retinas with rhodopsin, rod arrestin, PDE6β, M-opsin, cone arrestin, glutamine synthetase (GS), glial fibrillary acidic protein (GFAP), and actin (A) antibodies. Densitometric analysis of proteins normalized to actin (B). Data are mean ± *SEM* (*n* =4), *P < 0.001. *Ldha^flox/flox^* and *^ret^Ldha^−/−^* mouse retina sections were stained for rhodopsin, rod arrestin, PDE6β, M-opsin, S-opsin, cone arrestin/PNA, GS, and GFAP (C) antibodies. ROS, rod outer segments; POS, photoreceptor outer segments; RIS, rod inner segments; ONL, outer nuclear layer; OPL, outer plexiform layer; INL, inner nuclear layer; IPL, inner plexiform layer; GCL, ganglion cell layer. Scale bar = 50 μm. Altered S-opsin gradient in *^ret^Ldha^−/−^* mouse retina. Quantification of the number of S-opsin-positive and M-opsin-positive cones in the dorsal and ventral regions of the retina counted starting from the optic nerve head (D). Data are mean ± *SEM* (*n* = 6). *P < 0.001.

Examination of 4-week-old *Ldha^flox/flox^* and *^ret^Ldha^−/−^* mouse retinas showed no significant differences in the levels of rod photoreceptor markers, rhodopsin, rod arrestin, phosphodiesterase-6β (PDE6β), and cone-photoreceptor markers, M-opsin, and cone arrestin (Fig. 5A, B). The levels of Müller cell markers GS, and glial fibrillary acidic protein (GFAP) were significantly higher in *^ret^Ldha^−/−^* mice than in *Ldha^flox/flox^* mice (Fig. 5A, B). Immunohistochemistry analysis of *Ldha^flox/flox^* and *^ret^Ldha^−/−^* mouse retinas further confirmed that there was no significant difference in the expression of rhodopsin, rod arrestin, PDE6β, M-opsin, S-opsin, and cone arrestin (Fig. 5C). Consistent with the immunoblot analysis, the expression of GS and GFAP were higher in *^ret^Ldha^−/−^* mouse retinas than in *Ldha^flox/flox^* mouse retinas (Fig. 5C). Even though we found a loss of rod function in 28-week-old *^ret^Ldha^−/−^* mice (Fig. 4), there was no significant difference in the levels of rhodopsin, rod arrestin, PDE6β, M-opsin, cone arrestin, Glut1, HK2, PKM2, pPKM2, PKM1, and GS between *^ret^Ldha^−/−^* and *Ldha^flox/flox^* mice (Figure S1). The GFAP levels were still significantly higher in 28-week-old *^ret^Ldha^−/−^* mice than in *Ldha^flox/flox^* mice (Figure S1).

### Loss of dorsal–ventral patterning of cone-opsin gradient in retLdha−/− mice

In general, S-opsin-positive cones are largely expressed in the ventral area of the retina compared with the dorsal areas, whereas M-opsin-positive cones are dispersed in both dorsal and ventral areas, but have slightly higher distribution in the dorsal region of the retina (17). We found a significantly increased number of S-opsin-positive cones in the dorsal region and a significantly reduced number of S-opsin-positive cones in the ventral region in *^ret^Ldha^−/−^* mice compared with *Ldha^flox/flox^* mice (Fig. 5D, Figure S2). M-opsin expression was not altered in dorsal or ventral regions in *^ret^Ldha^−/−^* mice (Fig. 5D, Figure S2). These findings suggest that LDHA regulates the dorsal–ventral patterning of the S-opsin gradient.

### Effect of loss of LDHA on retinal metabolism

**Fig. 6.**
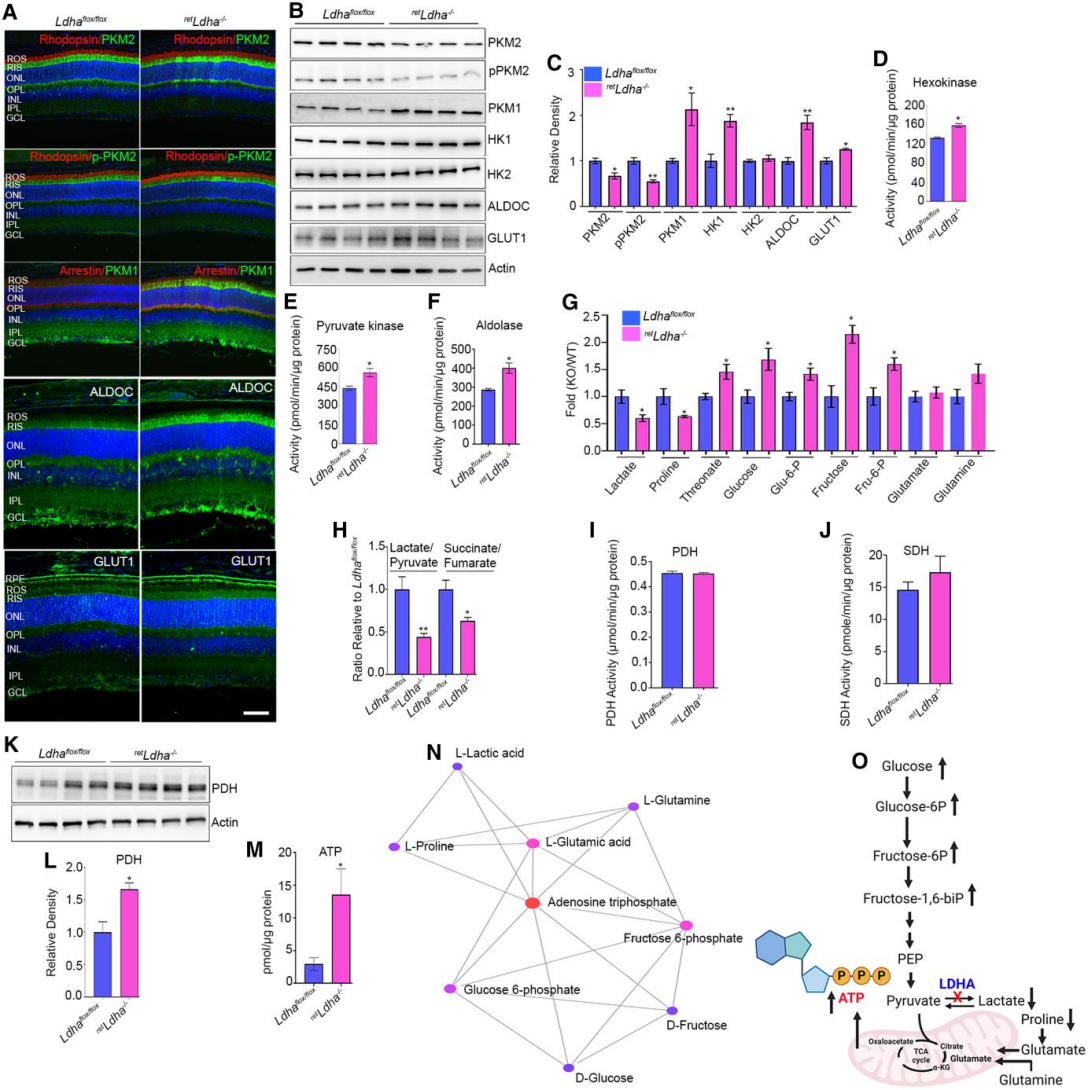
Effect of loss of LDHA on retinal metabolism. Four-week-old *Ldha^flox/flox^* and *^ret^Ldha^−/−^* mouse retina sections were stained for rhodopsin/PKM2, rhodopsin/pPKM2, rod arrestin/PKM1, aldolase C (ALDOC), and Glut1 (A) antibodies. Immunoblot analysis of 4-week-old *Ldha^flox/flox^* and *^ret^Ldha^−/−^* mouse retinas with PKM2, pPKM2, PKM1, HK1, HK2, ALDOC, GLUT1, and actin (B) antibodies. Densitometric analysis of proteins normalized to actin (C). Data are mean ± *SEM* (*n* = 4). *P < 0.05; **P < 0.001. Hexokinase activity (D, *n = 8*, *P < 0.0001), pyruvate kinase activity (E, *n = 11*, *P < 0.0032), aldolase activity (F, *n = 4*, *P < 0.012), PDH activity (I, *n = 6*, *P < 0.0001), SDH activity (J, *n = 6*, *P < 0.029) and ATP levels (M, *n = 4*, *P < 0.039) were measured in *Ldha^flox/flox^* and *^ret^Ldha^−/−^* mice. Data are mean ± *SEM.* Immunoblot analysis of *Ldha^flox/flox^* and *^ret^Ldha^−/−^* mouse retina samples with PDH and actin antibodies (K) and panel L represent the densitometric analysis of PDH/actin. Data are mean ± SEM, n = 4, *P < 0.01. Steady-state level retinal metabolites were measured from 8-week-old *Ldha^flox/flox^* and *^ret^Ldha^−/−^* mouse retina (G). Data are mean ± *SEM* (*n* =4), *P < 0.05. Lactate/pyruvate and succinate/fumarate ratio of *Ldha^flox/flox^* and *^ret^Ldha^−/−^* mouse retina (H, *n = 4*. **P < 0.01, *P < 0.02). Panel N represents the interaction analysis between metabolites in *^ret^Ldha^−/−^* mouse retinas whereas panel O represents the involvement of identified metabolites in glycolysis and the Krebs cycle.

In 4-week-old *^ret^Ldha^−/−^* mice, we found significantly decreased expression of PKM2 and its phosphorylation, whereas the expression of PKM1, aldolase C (ALDOC), and Glut 1 was higher than in *Ldha^flox/flox^* mice (Fig. 6A). Immunoblot analysis further confirmed significantly decreased levels of PKM2 and its phosphorylation, and increased levels of PKM1, ALDOC, and Glut1 in *^ret^Ldha^−/−^* mice compared with *Ldha^flox/flox^* mice (Fig. 6B, C). We found significantly increased levels of hexokinase 1 but not hexokinase 2 (Fig. 6B, C), and increased activities of hexokinase (Fig. 6D), pyruvate kinase (Fig. 6E), and aldolase (Fig. 6F) in *^ret^Ldha^−/−^* mice compared with *Ldha^flox/flox^* mice. To determine the effect of loss of LDHA in the retina, we measured 48 metabolites in 8-week-old *Ldha^flox/flox^* and *^ret^Ldha^−/−^* mice (Table S1). The analysis indicated significantly decreased levels of lactate and proline and increased levels of threonate, glucose, glucose 6-phosphate, fructose, and fructose 6-phosphate in *^ret^Ldha^−/−^* mice compared with *Ldha^flox/flox^* mice (Fig. 6G). There was no significant difference in the levels of glutamate between *^ret^Ldha^−/−^* and *Ldha^flox/flox^* mice (Fig. 6G). The levels of glutamine were higher in *^ret^Ldha^−/−^* mice than in *Ldha^flox/flox^* mice; however, the difference was not statistically significant (Fig. 6G). Our metabolite data show that there was a statistically significant decrease in the lactate/pyruvate and also succinate/fumarate ratio in *^ret^Ldha^−/−^* mice compared with *Ldha^flox/flox^* mice (Fig. 6H). We found no significant difference in the activities of PDH and SDH between *^ret^Ldha^−/−^* mice and *Ldha^flox/flox^* mice (Fig. 6I, J). However, we found significantly increased protein levels of PDH in *^ret^Ldha^−/−^* mice compared with *Ldha^flox/flox^* mice (Fig. 6K, L). Under these conditions, we found that steady-state ATP levels are three times higher in *^ret^Ldha^−/−^* mice compare to *Ldha^flox/flox^* mice (Fig. 6M). We performed a metabolite-metabolite interaction analysis to identify possible functional relationships between altered metabolites in *^ret^Ldha^−/−^* mouse retinas and the minimum network analysis of the analyzed steady-state metabolites and their interaction indicated that potential changes are centered on ATP (betweenness 8.16) (Fig. 6N, O, Table S2). The pathway analysis indicated impactful alterations in 1) Glu and Gln, 2) Ala, Asp, Glu, 3) Arg and Pro, 3) sucrose metabolism, and possible alterations in 4) Arg metabolism (Figure S3).

Examination of genes involved in glycolysis and the pentose phosphate pathway (PCR primers, Table S3) showed no significant difference in 6PDG, G6PD, and HIF1α expression in *^ret^Ldha^−/−^* and *Ldha^flox/flox^* mice, whereas the expression of PKM1 and ME1 were significantly higher and PKM2 was significantly lower in *^ret^Ldha^−/−^* mice than in *Ldha^flox/flox^* mice (Figure S4A). To determine whether loss of LDHA has any effect on mitochondria, we examined the expression of genes related to mitochondrial biogenesis (*Ppargc1a*, *Tfam*, *Nrf1*, and *Nrf2*), mitochondrial dynamics (*Drp1*, *Fis1*, *Opa1*, and *CypD*), and mitochondrial-encoded electron transport chain (*Cytb-CIII* and *COX1-CIV*) (PCR primers, Table S4). We found significantly increased expression of *Fis1* (mitochondrial dynamics) and *Cytb-CIII* and *COX1-CIV* (mitochondrial-encoded electron transport chain) genes in *^ret^Ldha^−/−^* mice compared with *Ldha^flox/flox^* mice (Figure S4B).

### Effect of loss of LDHA in Müller cells on retinal function

**Fig. 7.**
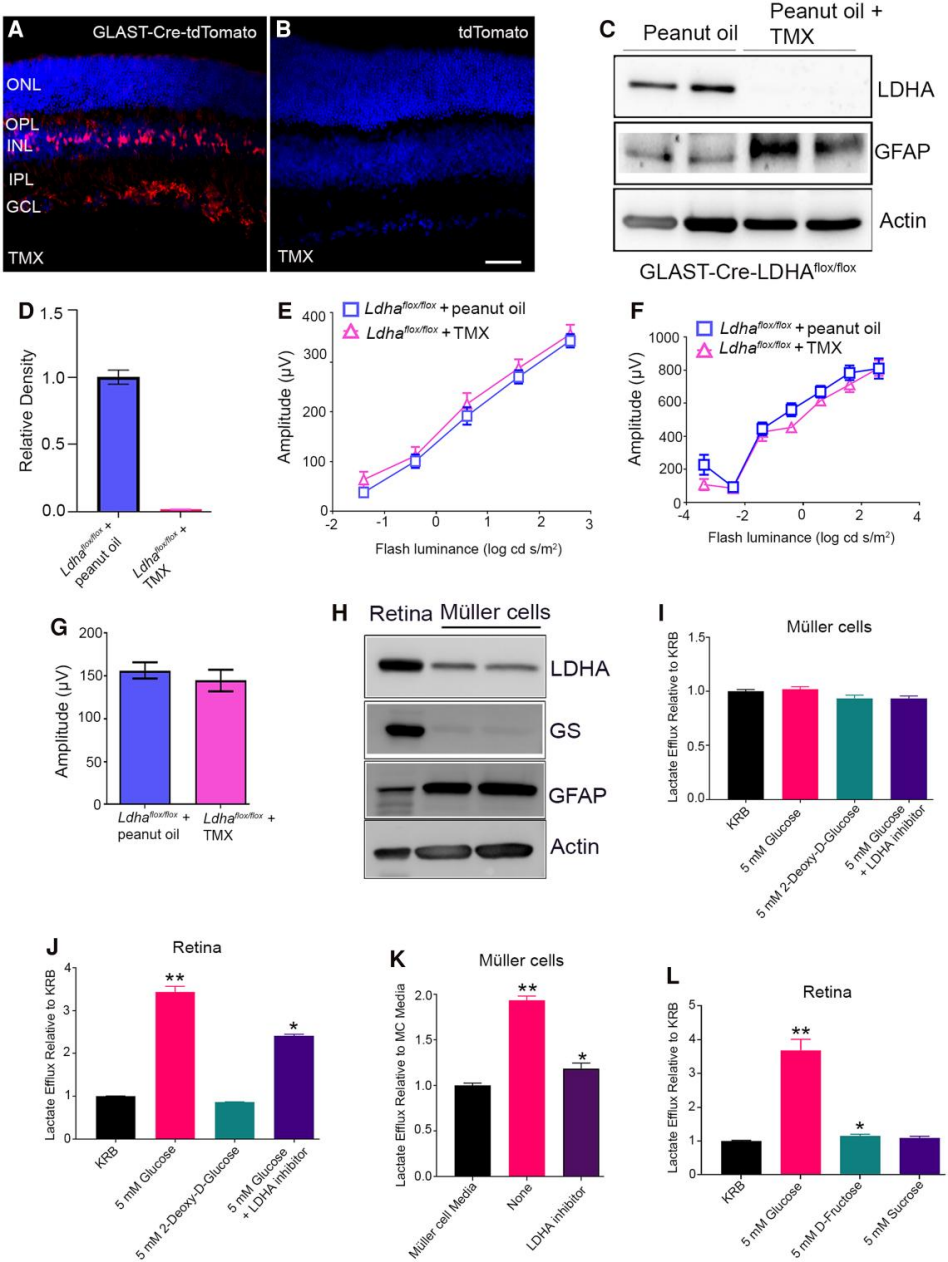
Effect of loss of LDHA in Müller cells on retinal function and lactate efflux in Müller cells. GLAST-tdTomato mice were induced with TMX (A) and without TMX (B). Scale bar = 50 µm. Müller cell cultures were prepared for control and TMX-injected GLAST-Cre-LDHA^flox/flox^ mice and the proteins were immunoblotted with LDHA, GFAP, and actin (C) antibodies. Normalized levels of LDHA to actin (D). Data are mean ± *SEM* (*n* = 2). Peanut oil and TMX-injected GLAST-Cre-LDHA^flox/flox^ mice after 10-weeks were subjected to ERG and we measured scotopic a-wave (E), scotopic b-wave (F), and photopic b-wave (G) amplitudes. Data are mean ± *SEM* (*n* = 18). Retina and primary Müller cell-proteins were immunoblotted with LDHA, GS, GFAP, and actin antibodies (H). Lactate efflux from primary Müller cells (3.86 ×10^5 ^cells/ml) was carried out in the presence of Krebs–Ringer–Bicarbonate (KRB) buffer, 5 mM glucose, 5 mM 2-deoxy D-glucose (2DG) and 5 mM glucose plus 100 µM LDHA inhibitor (GSK2837808A, MCE MedChem Express). Lactate release was measured after 30 min using Lactate Reagent (Trinity Biotech Bray. Co, Wicklow, Ireland) (I). Data are mean ± *SEM* (*n* = 3). A single mouse retina from C57Bl6 mice was incubated in the presence of KRB buffer, 5 mM glucose, 5 mM 2-deoxy D-glucose (2DG), and 5 mM glucose plus 100 µM LDHA inhibitor and measured lactate release after 30 min (J). Data are mean ± *SEM* (*n* = 3). **P < 0.0001, significance between KRB and 5 mM glucose; * *P* < 0.0017, significance between 5 mM glucose and 5 mM glucose plus 100 µM LDHA inhibitor. Primary Müller cells in a culture media containing 25 mM glucose grown in the presence and absence of 100 µM LDHA inhibitor. Twenty-four hours later, lactate release in the medium was measured as described above (K). Data are mean ± *SEM* (*n* = 3). **P < 0.0001, significance between Müller cell media and media containing Müller cells; * *P* < 0.0001, significance between media containing Müller cells and media containing Müller cells in the presence of 100 µM LDHA inhibitor. A single mouse retina from C57Bl6 mice was incubated in the presence of KRB buffer, 5 mM glucose, 5 mM D-fructose and 5 mM sucrose, and measured the released lactate after 30 min (L). Data are mean ± *SEM* (*n* = 4). **P < 0.0002, significance between KRB and 5 mM glucose; * *P* < 0.02 significance between KRB and 5 mM fructose.

We showed in Fig. 2C, very little expression of LDHA in Müller cells. To determine the functional role of LDHA in Müller cells, first, we determined Cre-recombinase expression in Müller cells by crossing a tamoxifen-inducible glial high-affinity glutamate transporter (GLAST) promoter-Cre line with an Ai9 reporter line with a *loxP*-flanked STOP cassette preventing transcription of a CAG promoter-driven red fluorescent protein variant (tdTomato) inserted into the *Gt(ROSA)26Sor* locus. Mice were orally gavaged with peanut oil (control) or 1 mg tamoxifen (TMX in peanut oil) every alternate day for 3 days. Retinal cryosections were examined for tdTomato expression after three weeks. We found that mice carrying GLAST-Cre^ER^ and Ai9 reporter expressed robust tdTomato fluorescence following tamoxifen (TMX) induction (Fig. 7A). In the absence of TMX, tdTomato was not expressed (Fig. 7B). We used these mice to generate Müller cell-specific deletion of the LDHA isoform. GLAST-Cre-*Ldha ^flox/flox^* mice were orally gavaged with either peanut oil (control) or 1 mg TMX every alternate day for 3 days (3 mg total). Since, the expression of LDHA in Müller cells *in vivo* is very low and is very difficult to examine the deletion of LDHA *in vivo*, three weeks after TMX administration, Müller cell cultures were prepared from adult mice and examined the expression and deletion of LDHA. Our results indicated the expression of LDHA in peanut oil-treated mice (Fig. 7C) but is deleted in TMX-treated mice (Fig. 7C, D). We found increased expression of GFAP in mouse Müller cells lacking LDHA compared to Müller cells containing LDHA in culture. TMX or peanut-gavaged GLAST-Cre-*Ldha ^flox/flox^* mice after 10 weeks were examined for function. There was no significant difference in scotopic a-wave (Fig. 7E), scotopic b-wave (Fig. 7F), and photopic b-wave (Fig. 7G) amplitudes between control and TMX-treated GLAST-Cre-*Ldha ^flox/flox^* mice.

Müller cells do not proliferate after 14 days; hence, we prepared Müller cells from wild-type C57Bl6 P7 pups and found the expression of LDHA in these cells (Fig. 7H), even though, we observe a very low level of its expression *in vivo* (Fig. 2C). These observations suggest that in culture, primary Müller cells might substantially change their properties. We examined lactate efflux to determine whether LDHA in Müller cells is functional. When we incubated primary Müller cells in the presence of 5 mM glucose, 5 mM 2-deoxy-glucose (2-DG), and glucose plus 100 µM LDHA inhibitor, we found no release of lactate in the presence of glucose with comparable data between these three groups (Fig. 7I). These observations suggest that primary Müller cells do not generate lactate from glucose. However, when we incubated mouse retinas in the presence of glucose, 2DG, and glucose with an LDHA inhibitor, we found a significant release of lactate in the presence of glucose, an absence of lactate release in the presence of 2DG, and a significantly decreased release of lactate in the presence of glucose and LDHA inhibitor (Fig. 7J). However, in regular culture conditions (media contains 25 mM glucose) following 24 hours post-plating of Müller cells, we found significantly higher levels of lactate in the culture media compared to Müller cell media (Fig. 7K). In the presence of an LDHA inhibitor, lactate levels in the media were significantly decreased compared to culture media (Fig. 7K). These observations suggest that Müller cells in culture may change their properties and become more glycolytic. Our steady-state metabolite analysis also indicated that fructose levels are significantly higher in mouse retinas lacking LDHA. We carried out lactate efflux in the presence of fructose, glucose, and sucrose. Our results indicate a significant release of lactate in the presence of fructose compared to buffer, whereas lactate efflux was higher in the presence of glucose (Fig. 7L). There was no lactate efflux in the presence of sucrose (Fig. 7L).

## Discussion

LDHA converts pyruvate to lactate utilizing NADH as a cofactor, whereas LDHB converts lactate to pyruvate utilizing NAD as a cofactor (18). Extensive studies on LDHA have been carried out in tumors; to date, there are very limited studies on LDHA in the retina. In the present study, we report that LDHA is predominantly localized to the outer retina, whereas LDHB is expressed in the inner retina. LDHA is known to be a hypoxia-inducible gene, whereas hypoxia represses LDHB in primary rat and chick retina cultures (12). Thus, LDHA and LDHB expression could be related to the oxygen gradient in the retina. LDHB is important in maintaining a high pyruvate level, and its expression is related to mitochondrial function and energy production. The rod photoreceptors have huge mitochondria, yet LDHB is localized to the inner retina.

Gene regulatory networks are conventionally identified through scRNAseq, but this technique will induce changes to gene expression that result from tissue dissociation and delays during cell sorting. To overcome these limitations, we employed a novel and innovative *in vivo* method to isolate actively translating mRNAs from the rod, cone, Müller, and RPE cells. Our data show that LDHA is predominantly expressed in rods and cones, whereas LDHB is predominantly expressed in Müller cells and RPE, further supporting that PR-made lactate is utilized by RPE and Müller cells (8). We also observed that loss of LDHA in the retina increased the expression of genes that regulate mitochondrial dynamics and mitochondrial-encoded electron transport. Our studies appear to indicate that LDHA and LDHB isoform expression is developmentally regulated.

In a previous study, a marked reduction of the outer segment length was observed when LDHA is targeted by a sequence-specific shRNA in the mouse retina (3). In the present study, LDHA loss in the retina did not affect retina structure and function at four weeks. However, rod function declined at 28 weeks without a change in the expression levels of key photoreceptor proteins, suggesting a functional loss without structural defects. However, at 40 weeks of age, thinning of outer nuclear thickness was observed in *^ret^Ldha^−/−^* mice (Fig. 3I–L), suggesting photoreceptor degeneration resulting from loss of LDHA. Loss of LDHA resulted in the upregulation of GS, which converts glutamate into glutamine. Consistent with the increased GS expression, we also found increased glutamine levels. Glutamine acts as an antioxidant and protects the mouse brain from ischemic injury (19). In addition, GS has been shown to protect against neuronal degeneration in injured retinal tissues (20). The GS levels in 28-week-old LDHA KO mice were comparable to those of wild-type mice where we observed a loss of visual function. Further studies are needed to study the role of GS (Figure S5) in photoreceptor function.

We previously reported that PKM2, which is mainly involved in aerobic glycolysis, is expressed in the photoreceptor inner segments, whereas PKM1 is predominantly expressed in the inner retinal layers (15). Both PKM1 and PKM2 isoforms arise from a single gene as alternatively spliced products; exon 9 inclusion results in PKM1, and exon 10 inclusion results in PKM2 (21). In a single tissue, more than one isoform may be expressed, but individual cells may largely express one isoform (22). We (23) and others (3, 24) have shown that the deletion of PKM2 in photoreceptors resulted in the upregulation of PKM1. A novel observation made in this study is that loss of LDHA in the retina results in the upregulation of PKM1 in photoreceptors in the presence of PKM2. Our results show a 33% downregulation of PKM2 and its phosphorylation in LDHA KO mice, but this loss may not explain the upregulation of PKM1. These observations suggest that LDHA may have a regulatory role in the expression of PKM isoforms.

**Fig. 8.**
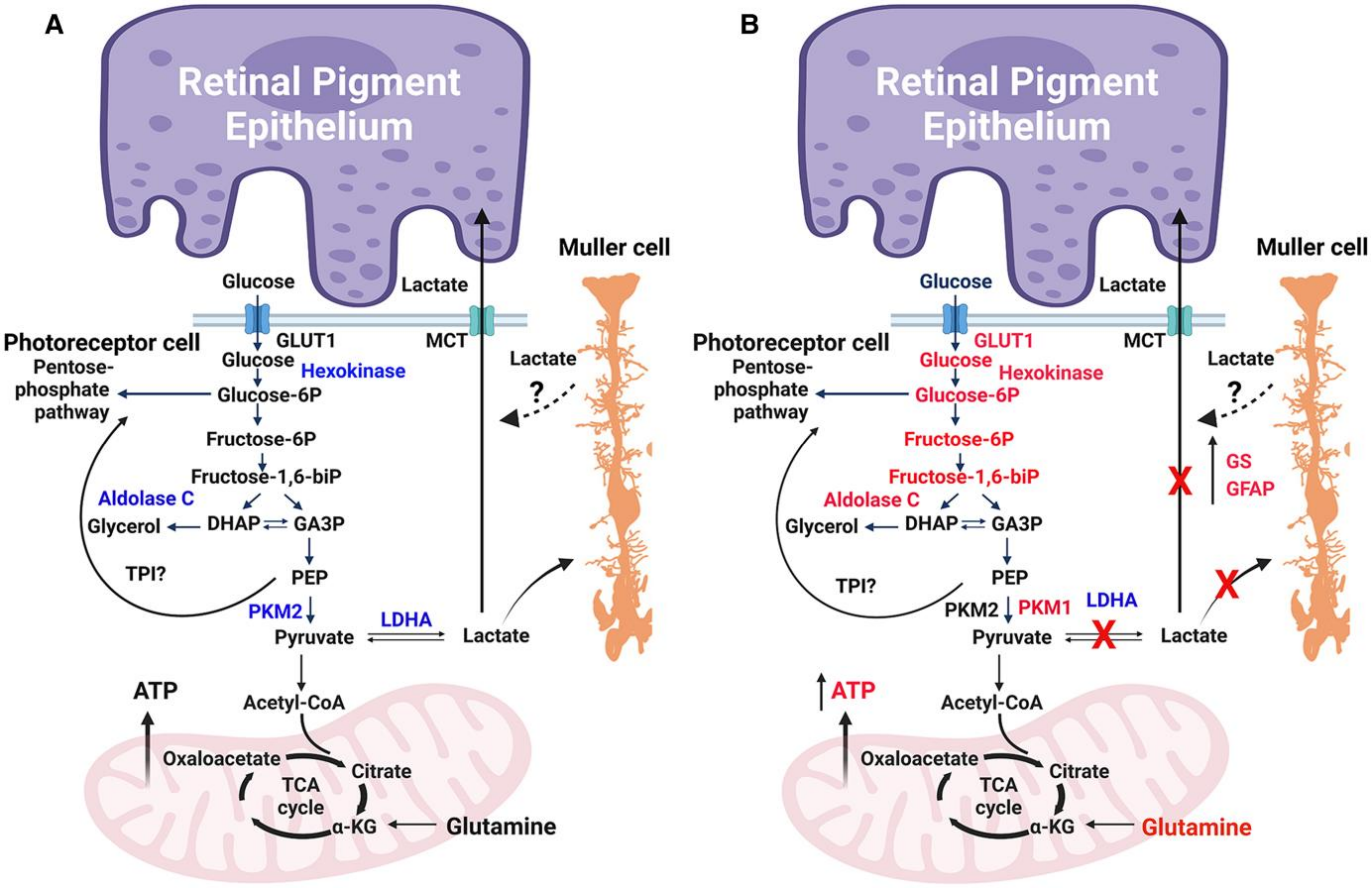
Working model of LDHA-mediated functions in the retina. Photoreceptor cells utilize glucose and produce large amounts of lactate in the presence of oxygen. This process is known as the “Warburg effect,” or aerobic glycolysis. RPE cells provide glucose to photoreceptor cells via glucose transporter 1 (GLUT1), and the glycolytic end-product pyruvate is converted to lactate by LDHA. The photoreceptor-generated lactate is transported to RPE and Müller cells via monocarboxylate transporters (MCT), where it is converted to pyruvate to fuel mitochondria for oxidative phosphorylation. In photoreceptor cells, PKM2 catalyzes the conversion of PEP to pyruvate. When PKM2 undergoes phosphorylation, PKM2 has a lower affinity for PEP and the accumulated PEP activates the pentose phosphate pathway via TPI. In yeast, decreased pyruvate kinase activity results in the accumulation of PEP which activates PPP via TPI (55). In cancer cells, reduced PKM2 activity has been shown to result in the increased anabolic synthesis of macromolecules via PPP (56). However, the activation of PPP via TPI has not been established in retina/photoreceptor cells. Fructose 1, 6-bisphosphate (FBP) is an allosteric activator of PKM2 and aldolase C cleaves FBP to generate DHAP and GA3P (A). When we deleted LDHA in the retina, we found increased levels of glycolytic intermediates and glycolytic enzymes (in red) and increased PKM1 expression with a decreased PKM2 expression; phosphorylated PKM2 favors oxidative phosphorylation (B). We found increased Müller cell resident glutamine synthetase expression, GFAP, and increased glutamine levels. Loss of LDHA resulted in decreased visual function, structural defects, and a loss of dorsal–ventral patterning of the cone-opsin gradient. Deletion of LDHA in Müller cells did not affect visual function, suggesting that lactate made in photoreceptors is transported to Müller cells. Glut1, glucose transporter 1; MCT, monocarboxylate transporter; PKM2, pyruvate kinase M2 isoform; PKM1, pyruvate kinase M1 isoform; DHAP, dihydroxy acetone phosphate; GA3P, glyceraldehyde 3-phosphate; PEP, phosphoenolpyruvate; LDHA, lactate dehydrogenase A; TPI, triose phosphate isomerase; GS, glutamine synthetase; GFAP, Glial fibrillary acidic protein; ATP, adenosine triphosphate; FBP, fructose 1,6-bisphosphate. This figure was created with BioRender.com.

Our studies also show that loss of LDHA in the retina resulted in increased glucose availability, which correlates with increased Glut1 expression. Increased activity and expression of glycolytic enzymes (hexokinase and PKM1) and increased ATP production further suggest that loss of LDHA favors OXPHOS (Fig. 8). Fructose 1,6-bisphosphate (FBP) is an allosteric activator of PKM2 when it binds to PKM2; it has a higher affinity for phosphoenolpyruvate (PEP) to generate pyruvate (6). FBP is cleaved by aldolase to generate glyceraldehyde 3-phosphate (G3P) and dihydroxyacetone phosphate (DHAP) (25). Phosphorylation of PKM2 blocks the binding of FBP, thereby increasing the accumulation of PEP, which activates the PPP for cellular anabolism (6). Both DHAP and G3P are 3-carbon sugars, which are crucial molecules for the synthesis of glycerol, the backbone of lipids. Glycerol synthesis requires glycerol-3-phosphate dehydrogenase 1 (GPD1) enzyme. Isolation rod photoreceptors by Translating Ribosome Affinity Purification (TRAP) followed by RNA sequencing show very low levels of *Gpd1*, however, photoreceptors express higher levels of *Gpd1*-like (*Gpd1l*) (Figure S6). GPD1L has been shown to perform the same functions as GPD1 in other cell types (26). Therefore, it appears GPD1L is the predominant isoform of GDP1 in rod photoreceptors and its active translation seems to suggest a role in glycerol synthesis. In the lipid bilayer, one molecule of rhodopsin requires 60 molecules of a phospholipid for its function (27), suggesting that a high rate of glycerol/phospholipid synthesis is required in photoreceptors. Defects in lipid synthesis lead to visual impairments, which ultimately lead to blindness (28). In the present study, decreased levels of PKM2 and its phosphorylation and increased PKM1 expression, and increased pyruvate kinase activity in retinas lacking LDHA further confirmed the switching of cells from aerobic glycolysis to OXPHOS. The increased aldolase C expression, which limits the availability of FBP to activate PKM2 in mice lacking LDHA may generate glycerol for phospholipid biosynthesis. This probably explains no change in rhodopsin expression and levels in LDHA KO mouse retinas.

Interestingly, in LDHA KO mouse retinas, we observed a significant decrease in the Lactate/Pyruvate and also in the Succinate/Fumarate ratio. These observations suggest that the reducing potential in the retina (probably in the rods) is much lower than normal (NADH/NAD). Under these conditions, we found steady-state ATP levels are at least three times higher in LDHA KO mice than in wild-type mice. One way that these things could happen is if the rods had more mitochondria or if mitochondria were more active at oxidizing NADH to NAD than normal. Consistent with this idea, we found increased expression of transcripts encoding mitochondrial-encoded electron transport chain enzymes and complexes, mitochondrial dynamics, and significantly increased protein levels of PDH in LDHA KO mouse retinas compared to wild-type. Further studies are necessary to examine how the loss of LDHA affects mitochondrial dynamics.

The lack of structural phenotype in mouse retina lacking LDHA suggests that there could be an alternative pathway that provides NADPH needed for lipid biosynthesis. The increased GS activity may be involved in the alternative NADPH production. We know that malate derived from glutamine in the mitochondria shuttles to the cytosol to produce NADPH (29). The major source of NADPH is through the pentose phosphate pathway (PPP), but under transient nutrient shortage conditions, the photoreceptor mitochondria provide an alternative metabolic pathway for NADPH generation (30). GS has been shown to protect against neuronal degeneration in injured retinal tissues (20). The absence of functional and structural phenotypes in mice lacking LDHA at early time points could be due to increased expression of GS. However, at 28 weeks of age, we found no increase in GS levels in mice lacking LDHA, and photoreceptor degeneration was observed at 40 weeks of age. We did not observe an increase of GS in rods (Figure S5), suggesting that the increase we observed in mice lacking LDHA could be from Müller cells.

The controversial issue of Müller cells providing lactate to PR has not yet been resolved. Müller cells have been previously shown to metabolize glucose and provide lactate to the PR cells to support their oxidative phosphorylation (31). In contrast, Hurley and colleagues have proposed that PR cells provide lactate to Müller cells and RPE cells (5, 8). Glucose from the choroidal circulation passes through the RPE and is taken up by photoreceptor cells and, through glycolysis, is converted to pyruvate and then to lactate by LDHA (8). Then, the lactate is transported through lactate transporters (32, 33) to RPE and Müller cells, where it is converted to pyruvate to fuel their mitochondria (8). Thus, photoreceptors are more glycolytic, whereas RPE and Müller cells favor oxidative phosphorylation. Our studies show that LDHA expression is very low in Müller cells *in vivo*, however, when we isolated Müller cells from the retina, they show the expression of LDHA. Consistent with these observations the single cell Müller cell transcriptome from degenerating retina shows LDHA-like, and LDHA expression (34). Interestingly, we failed to observe lactate efflux in response to glucose in these cells, however, growing these cells in culture, releases lactate in the medium, suggesting that Müller cells in culture may change their properties and becomes more glycolytic. It has been shown *in vivo* that Müller glia does not complete glycolysis, but uses glucose to produce serine to support photoreceptors (35). In our study, 10-week-old mice after TMX administration, Müller cell-specific LDHA KO mice do not affect retinal function. These studies are consistent with a recently published study that Müller cell-specific deletion of LDHA KO mice has no morphological evidence of photoreceptor damage (35). The GLAST-Cre mouse line we have used to delete LDHA has been used in a majority of studies to determine gene function in Müller cells (36, 37). The only caveat with this line is that it targets both Müller glia and central nervous system astrocytes (37), yet we did not observe any retinal phenotype when we deleted LDHA using this Cre-line. Furthermore, our TRAP data show that Müller cells express higher levels of LDHB than LDHA, suggesting that lactate is converted to pyruvate in these cells. These observations support the earlier study that lactate produced in photoreceptor cells is transported to Müller cells to fuel mitochondria (8).

Another novel observation made in the present study is that LDHA regulates the dorsal–ventral patterning of the cone-opsin gradient. LDHA interconverts lactate to pyruvate in the cytosol and performs other functions in the nucleus: regulating cell cycle progression, activating SIRT1 by supplementing NAD^+^, and binding to single-stranded DNA to facilitating DNA replication by recruiting DNA polymerases (38–40). Thyroid hormone (TH) signaling is known to regulate the dorsal–ventral pattern of the cone-opsin gradient (41–43). Further studies are needed to examine the role of LDHA in TH signaling.

The reason for increased fructose levels in LDHA KO mouse retina is currently unknown. Fructose can be used to make glucose through gluconeogenesis. Our study also suggests that fructose may also serve as fuel for glycolysis, but not to the same extent as glucose. It is tempting to speculate that excess glucose may be converted to fructose, and such a possibility cannot be ruled out.

The increased glucose availability and switching of the cells from aerobic glycolysis to OXPHOS in mice lacking LDHA have therapeutic benefits. A recent study showed that photoreceptors of individuals with age-related macular degeneration (AMD) display increased expression of two key glycolytic enzymes, HK2 and PKM2, indicating that AMD is associated with a glucose shortage (44). Furthermore, oxidative stress has also been shown to increase the expression of PKM2 *in vitro* (45). It has also been shown that the ablation of PKM2 in the RP mouse model partially rescues the degenerating phenotype (46). Taken as a whole, our studies suggest that glucose availability for photoreceptors is critical for cell survival, and increasing availability during AMD progression by regulating the levels of LDHA may be therapeutically beneficial. In summary, our studies on LDHA unraveled unidentified unique functions in retinal cells.

## Materials and methods

### Antibodies

Polyclonal LDHA, LDHB, and aldolase C antibodies were purchased from Proteintech (Rosemount, IL). Rabbit polyclonal red/green cone opsin (M-opsin), S-opsin, cone arrestin, actin, and rabbit and mouse secondary antibodies were obtained from Millipore (Billerica, MA). Monoclonal 1D4 rhodopsin antibody was a kind gift from Dr. James F. McGinnis (University of Oklahoma Health Sciences Center). DAPI used for nuclear staining was procured from Invitrogen-Molecular Probes (Carlsbad, CA). Polyclonal pPKM2 (Y105), PKM2, PKM1, PDH, hexokinase 1, and hexokinase 2 antibodies were obtained from Cell Signaling (Danvers, MA). The monoclonal anti-arrestin antibody was a kind gift from Dr. Paul Hargrave (University of Florida, Gainesville). Polyclonal glial fibrillary acidic protein (*GFAP*) was purchased from Dako (Carpinteria, CA). Monoclonal GS antibody was purchased from Abcam (Cambridge, MA). The monoclonal Pde6β antibody was purchased from Santa Cruz Biotechnology (Dallas, TX).

### Animals

Our study followed the NIH Guide for the Care and Use of Laboratory Animals and the ARVO Statement for the Use of Animals in Ophthalmic and Vision Research. The Institutional Animal Care and Use Committee (IACUC) at the University of Oklahoma Health Sciences Center approved all protocols. The floxed LDHA (Stock No: 030112), Chx10-EGFP/Cre (Stock No: 005105), transgenic glial high-affinity glutamate transporter (GLAST-Cre^ER^), Rax-Cre^ER^ (Stock No: 025521), tdTomato reporter (Stock No: 007909), and RiboTag (Stock No: 011029) mice were purchased from the Jackson Laboratory (Bar Harbor, ME). The rhodopsin-Cre (i75-Cre) mice have been described earlier (47) and were provided by Dr. Ching-Kang Jason Chen (Baylor College of Medicine, Houston, TX). Tetracycline-inducible RPE-specific VMD2-Cre mice and cone opsin-Cre (human red/green pigment gene promoter) mice were provided by Dr. Yun Le (University of Oklahoma). All mice were screened for *rd1* and *rd8* mutations and were negative for these mutations. The eyes or retinas were harvested after CO_2_ asphyxiation. For metabolic experiments, mouse retinas were harvested under deep anesthesia, or retinas were removed after decapitation. These tissues were subjected to biochemistry or immunohistochemistry. To eliminate bias, mice of the same sex, age, and genetic strain were randomly assigned to each experimental group. Litters were mixed to prevent litter bias. Once mice were genotyped and provided with unique ear tag identifiers, cohorts were selected randomly by the principal investigator (Dr. Rajala), such that research personnel doing experiments were blinded and only knew the ear tag number.

### Immunohistochemistry and immunoblot analyses of retinas

Immunohistochemistry and immunoblot analysis were performed as previously described (48). In the current study, blots were incubated with LDHA (1:1,000), LDHB (1:1,000), hexokinase 1 (1:1,000), hexokinase 2 (1:1,000), Aldolase C (1:1,000), cone arrestin (1:1,000), pPKM2 (1:1,000), PKM2 (1:1,000), PKM1 (1:1,000), Pde6β (1:1,000), rhodopsin (1:1,000), rod arrestin (1:1,000), M-opsin (1:1,000), PDH (1:1,000) and actin (1:1,000) antibodies (Table S5) overnight at 4° C. The blots were then washed and incubated with HRP-coupled anti-mouse or anti-rabbit secondary antibodies (as appropriate) for 60 min at room temperature. After washing, blots were developed with enhanced SuperSignal West Dura Extended Duration Substrate (Thermo Fisher Scientific, Waltham, MA) and visualized using a Kodak Imager with chemiluminescence capability.

### Hexokinase activity

The hexokinase assay was carried out as described (49). Mouse retinas were lysed in 50 mM potassium phosphate, 2 mM DTT, 2 mM EDTA, and 20 mM NaF. The assay was carried out in the presence of mouse retinal lysate containing an enzyme buffer mixture [100 mM Tris–HCl (pH 8.0), 10 mM MgCl_2_, 0.5 mM EDTA, 10 mM ATP, and 0.2 mM NADP], 2 mM D-glucose, and 1 U glucose 6-phosphate dehydrogenase. The hexokinase activity was measured at 340 nm spectrophotometrically by monitoring the generation of NADPH.

### Pyruvate kinase enzyme assay

The lactate dehydrogenase (LDH) coupled enzyme assay was used to measure pyruvate kinase (PK) enzyme activity (15). The assay was carried out in the presence of mouse retinal lysate containing an enzyme buffer mixture (50 mM Tris–HCl [pH 7.4], 100 mM KCl, 5 mM MgCl_2_, 1 mM ADP, 0.5 mM PEP, and 0.2 mM NADH [reduced form of NAD^+^]) and 8 U of LDH with a reaction volume of 1.0 ml. The PK activity was measured spectrophotometrically by monitoring the reduction in the absorbance at 340 nm from the oxidation of NADH.

### Aldolase enzyme assay

The assay was carried out according to the method described earlier (50). The reaction mixture contained 50 mM Tris–HCl, 0.1 M potassium acetate buffer (pH 8.0), 0.1–5 mM FBP, 0.2 mM NADH, and a mixture of coupling enzymes, glycerol phosphate dehydrogenase, and triosephosphate isomerase. The reaction was initiated by the addition of retina lysate and we monitored the decrease in the absorbance at 340 nm from the oxidation of NADH.

### Affinity purification of actively translated mRNAs from the rod, Müller, and RPE cells

The RiboTag mice carrying HA epitope-tagged ribosomal protein (*Rpl22*^HA^) were bred with rod-specific rhodopsin-Cre, cone-opsin-Cre, Müller cell-specific Rax-Cre^ERT2^, and tetracycline-inducible RPE-specific VMD2-Cre to allow Cre-mediated HA epitope tagging of ribosomes to isolate actively translating mRNAs from rod photoreceptor, cone photoreceptor, Müller, and RPE cells. Rax-Cre^ERT2^ was induced with 1 mg tamoxifen every alternate day by gavage for 3 days, whereas VMD2-Cre was induced with doxycycline gavage at a dose of 0.4 mg/g (10 mg/mice) body weight for 2 days. We immunoprecipitated polyribosomes bound to mRNA (51) from those specific cell types and prepared RNA. The RNA is converted to cDNA and examined the expression of *Ldha*, *Ldhb*, *Rpl38*, and *β-actin* by qRT-PCR (Table S6.) We normalized the expression of *Ldha* and *Ldhb* to *Rpl38* and *β-actin*. RNA samples from retina and rod-Cre/TRAP samples were subjected to RNA sequencing at MedGenome, Inc (Foster City, CA). The RNAseq data were used to examine the expression of *Gpd1* and *Gpd1l* in rod photoreceptor cells (Figure S6).

### Establishment of primary Müller cell cultures—

We have employed the Müller cell isolation protocol according to the method described (52) with the following modifications. Before harvesting the eyes, we sterilized all the dissection tools and disinfected the tabletop dissecting microscope and inverted microscope. We filtered DMEM, FBS, glutamine, and Pen/Step. We also filtered collagens and prepared 1% Pen/Step in 1 X PBS. We have also sterilized aluminum foil with 70% ethyl alcohol (ETOH), cleaned the Biosafety Cabinet (BSC) with 70% ETOH, and switched the UV light at least 15 min before bringing the mouse eyes into the BSC. On day one, we decapitated the P5 C57Bl6 pups and with the aid of a microscope, we made an incision on the cornea and stretched the muscle with forceps. We then enucleated the eye and placed it on a petri dish containing 1 X PBS. We wiped all the tools with a disinfectant cloth (repeated this step for every mouse eye). We have used five pups (10 eyes) for Müller Cell Primary Cultures. This procedure was conducted outside of the BSC. Once the dissection is complete, we wiped the petri dish containing eyes with 70% ETOH and then transported this dish to BSC. The rest of the procedure described was conducted in the culture facility. Transferred the eyeballs into a clean petri dish and washed the eyeballs five times with 1 X PBS and carefully aspirate the 1 X PBS at each wash. To another petri dish, added 5 ml of 1% Pen/Strep and transferred the eyeballs into this dish, and incubated the eyeballs at room temperature for 30 min. Again, washed the eyes two times with 1 X PBS and took a new petri dish and added 10 ml DMEM containing Glutamine and Pen/Strep, and wash with this medium two times. After washing, added 10 ml of the same medium, and the petri dish was wrapped with sterile aluminum foil and kept at room temperate overnight in dark. On day two, the eyeballs were transferred into a new petri dish containing 10 ml DMEM containing Glutamine and Pen/Step and washed with the same medium two times. In a 15 ml Falcon tube, added 4 ml 1 X PBS containing collagenase and trypsin, transferred the eyeballs into this tube and incubated at 37° C in a BOD incubator (95% O_2_ and 5% CO_2_) for 60 min with a loose cap. After incubation, the eyeballs were washed 3 times with DMEM containing 10% FBS, Glutamine, and Pen/Step. Under a microscope, the retinas were removed from the rest of the eye and collected the retina into a 1.5 ml Eppendorf tube containing 1 ml of DMEM containing 10% FBS, Glutamine, and Pen/Strep. The retinas were triturated up and down with a 1 ml pipette tip 20 times to make a uniform dispersion without any chunks of the retina. We then transferred the suspension into a 15 ml Falcon tube containing 4 ml DMEM containing 10% FBS, Glutamine, and Pen/Strep. We mixed the suspension with a Pastures pipette up and down 10 times to make a uniform suspension. The suspension was transferred to a 60 mm petri dish, and incubated culture in BOD incubated for 4 days. After 2 days, we added 2 ml DMEM containing 10% FBS, Glutamine, and Pen/Strep on top of the 4 ml original medium. On day 4, the culture was examined under a microscope and we found Müller cells firmly attached to the plate. We then replaced the medium with 5 ml DMEM containing 10% FBS, Glutamate, and Pen/Strep. Experiments were conducted on cells after three to four passages.

### Statistical analysis

We estimated the sample size by power analysis (53). Data were subjected to appropriate statistical evaluation to determine significant changes using GraphPad Prism 7.3 software. We did not exclude any sample from the statistical analysis. We used several statistical methods depending on the type of experiments. Before determining the statistical analysis, we performed a series of normality tests on the data (Anderson–Darling test, D’Agostino & Pearson test, Shapiro–Wilk test, Kolmogorov–Smirnov test) to determine whether the data were normally distributed in a Gaussian manner or not normally distributed. If the data were not normally distributed, we performed the unpaired non-parametric test to compare the two groups. We ran multiple Mann–Whitney U tests on the data. To correct for multiple comparisons, we controlled the false discovery rate by setting Q = 1% and used the two-stage step of the method of Benjamini, Krieger, & Yekutieli. For the normally distributed data, we performed a parametric test to compare the two groups. We ran Welch's correction to determine statistical significance. The resulting *P* values were used to deduce significance. We also used one-way ANOVA to determine whether there were any statistically significant differences between the means of three or more independent (unrelated) groups. We used a two-way ANOVA to estimate how the mean of a quantitative variable change according to the levels of two categorical variables.

### Other methods

Electroretinography (ERG) was carried out as described (23). The pyruvate dehydrogenase (PDH) activity assay kit was obtained from the Biomedical Research Service, University at Buffalo State University of New York. The PDH enzyme activity assay is based on the reduction of the tetrazolium salt INT in NADH-coupled reaction to formazan, which exhibits an absorption maximum at 492 nm (ε = 18 mM^−1^ cm^−1^) and allows for measurement of PDH activity in retinal tissue. The SDH enzyme activity was measured using a kit from Sigma. ATP concentration was determined using an EnzyLight ATP Assay Kit from BioAssay Systems (Hayward, CA). OptiPrep density gradient centrifugation was used to isolate photoreceptors with attached or broken inner segments (15). Lactate efflux was carried out according to the method described earlier (9). Steady-state metabolites were measured as we described recently (54). We performed a metabolite-metabolite interaction analysis using MetaboAnalyst 5.0 (https://www.metaboanalyst.ca/) to identify possible functional relationships between altered metabolites in *^ret^Ldha^−/−^* mouse retinas as described (54).

## Supplementary Material

pgad038_Supplementary_Data

## Data Availability

All data are included in this article and/or Supplementary material.
