# Supplementary material for: The function of lactate dehydrogenase A in retinal neurons: implications to retinal degenerative diseases
Source: PNAS Nexus. 2023 Feb 3;2(3):pgad038. doi: 10.1093/pnasnexus/pgad038 (PMC9991461; doi:10.1093/pnasnexus/pgad038)
Supplement: pgad038_Supplementary_Data [file pgad038_supplementary_data.pdf]

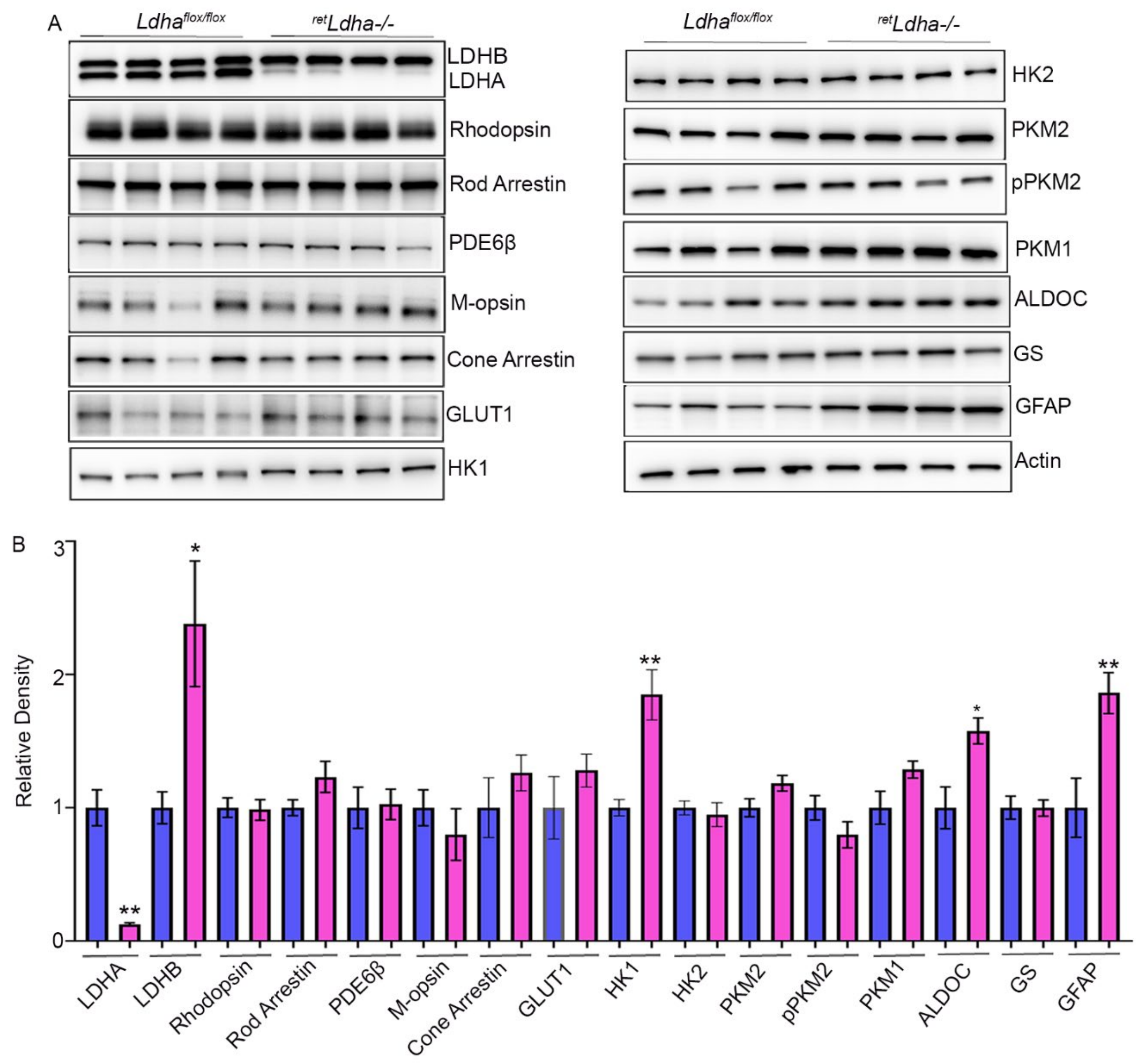

Figure S1. Twenty-eight-week-old *Ldha*$^{flox/flox}$ and ^{ret}*Ldha*$^{-/-}$ mouse retinal proteins were immunoblotted with LDHA, LDHB, rhodopsin, rod arrestin, PDE6β, M-opsin, cone-arrestin, GLUT1, HK1, HK2, PKM2, pKM2, PKM1, ALDOC, GS, GFAP, and actin antibodies (**A**). Densitometric analysis of protein normalized to actin (**B**). The data are mean ± *SEM* (*n*=3). **p*<0.05, ***p*<0.001.

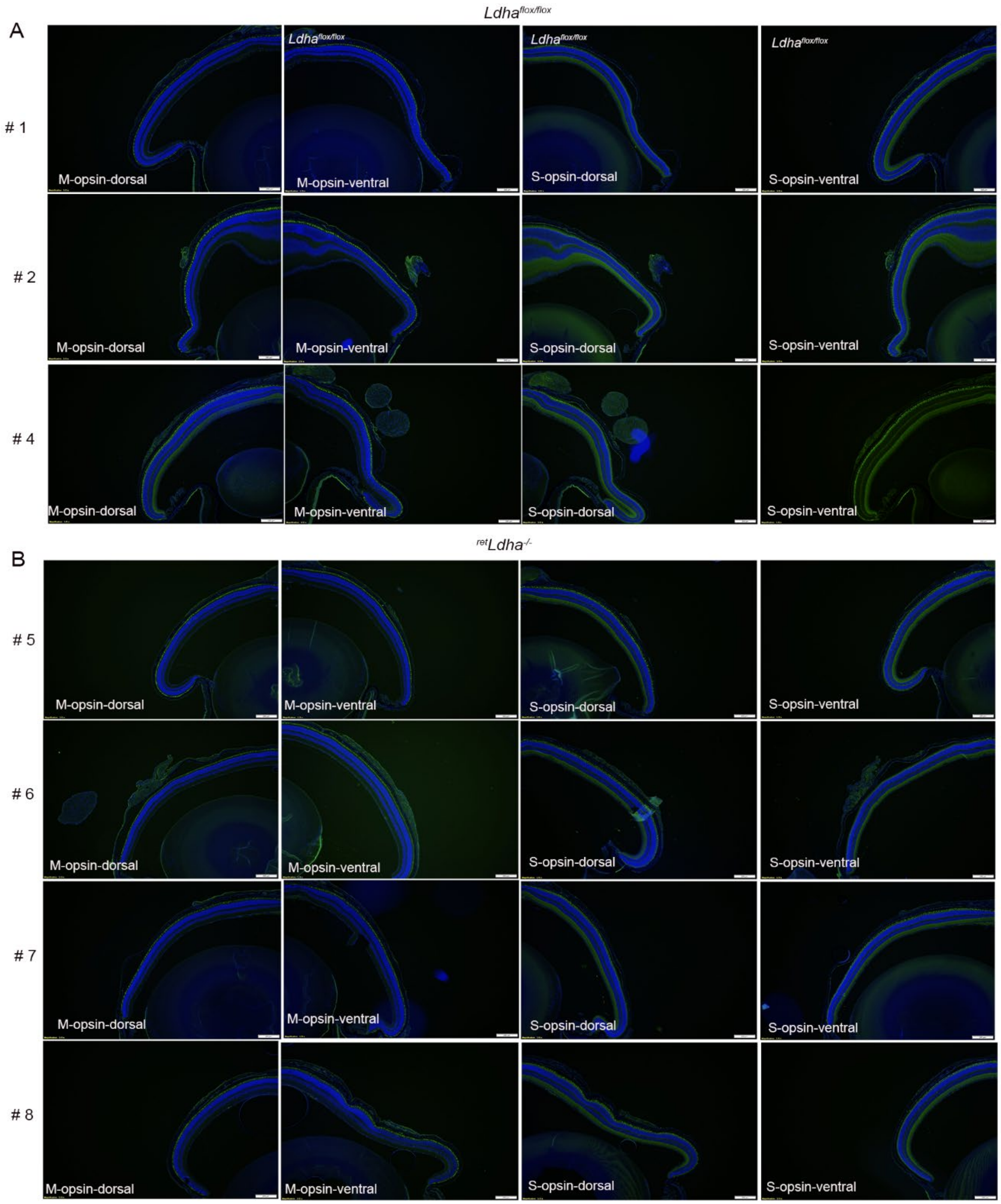

Figure S2. LDHA regulates the dorsal-ventral gradient of S-opsin- and M-opsin-positive cones. Mouse retina sections from *Ldha*$^{flox/flox}$ and ^{ret}*Ldha*$^{-/-}$ mice were stained with S-opsin and M-opsin antibodies. The number of S-opsin-positive and M-opsin-positive cones in the dorsal and ventral regions of the retina was counted starting from the optic nerve head.

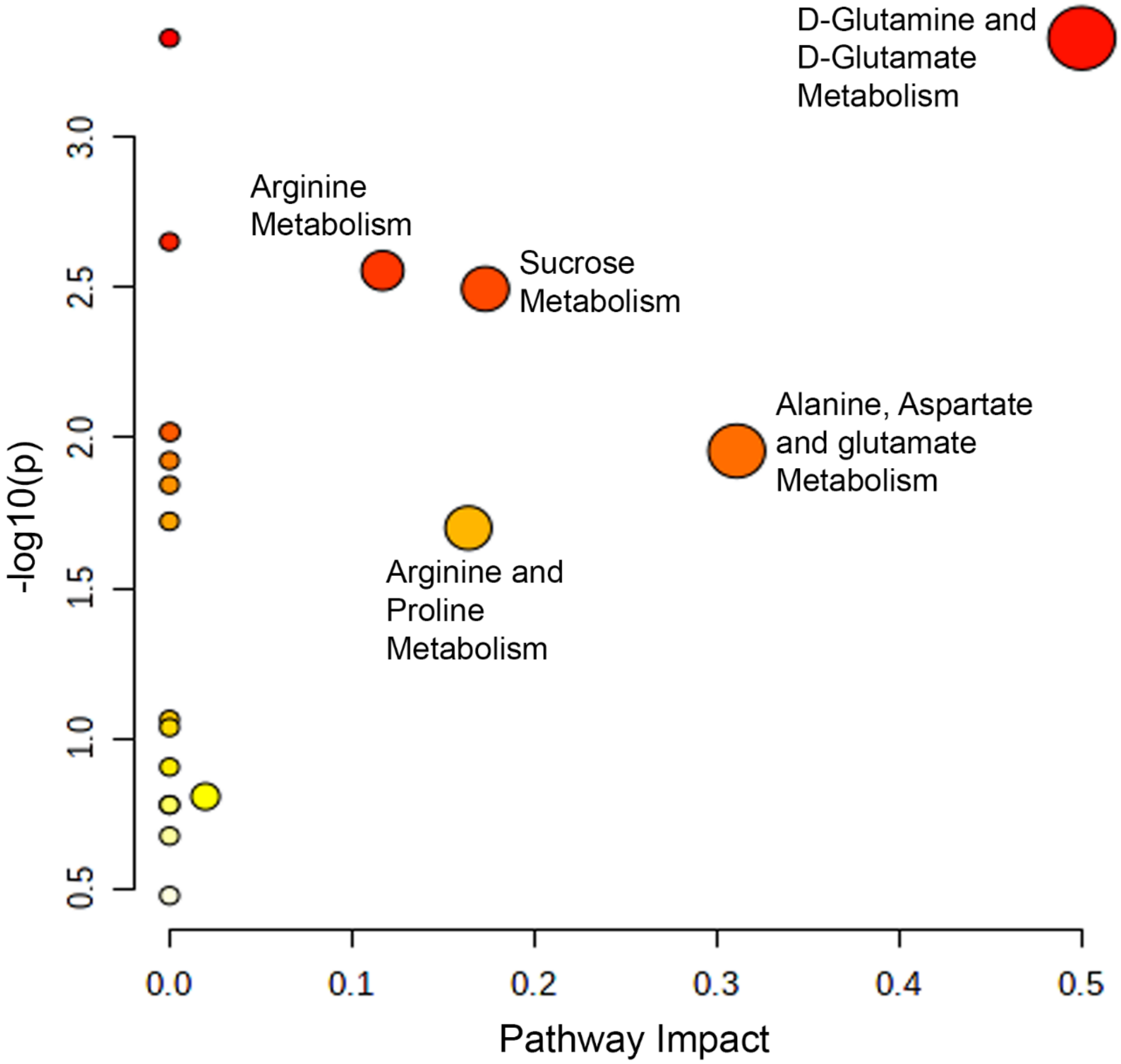

Figure S3. Altered metabolic pathways in ^{ret}*Ldha*$^{-/-}$ mouse retina. Steady-state level retinal metabolites were measured from 2-month-old ^{ret}*Ldha*$^{-/-}$ mouse retina and data were subjected to network analysis.

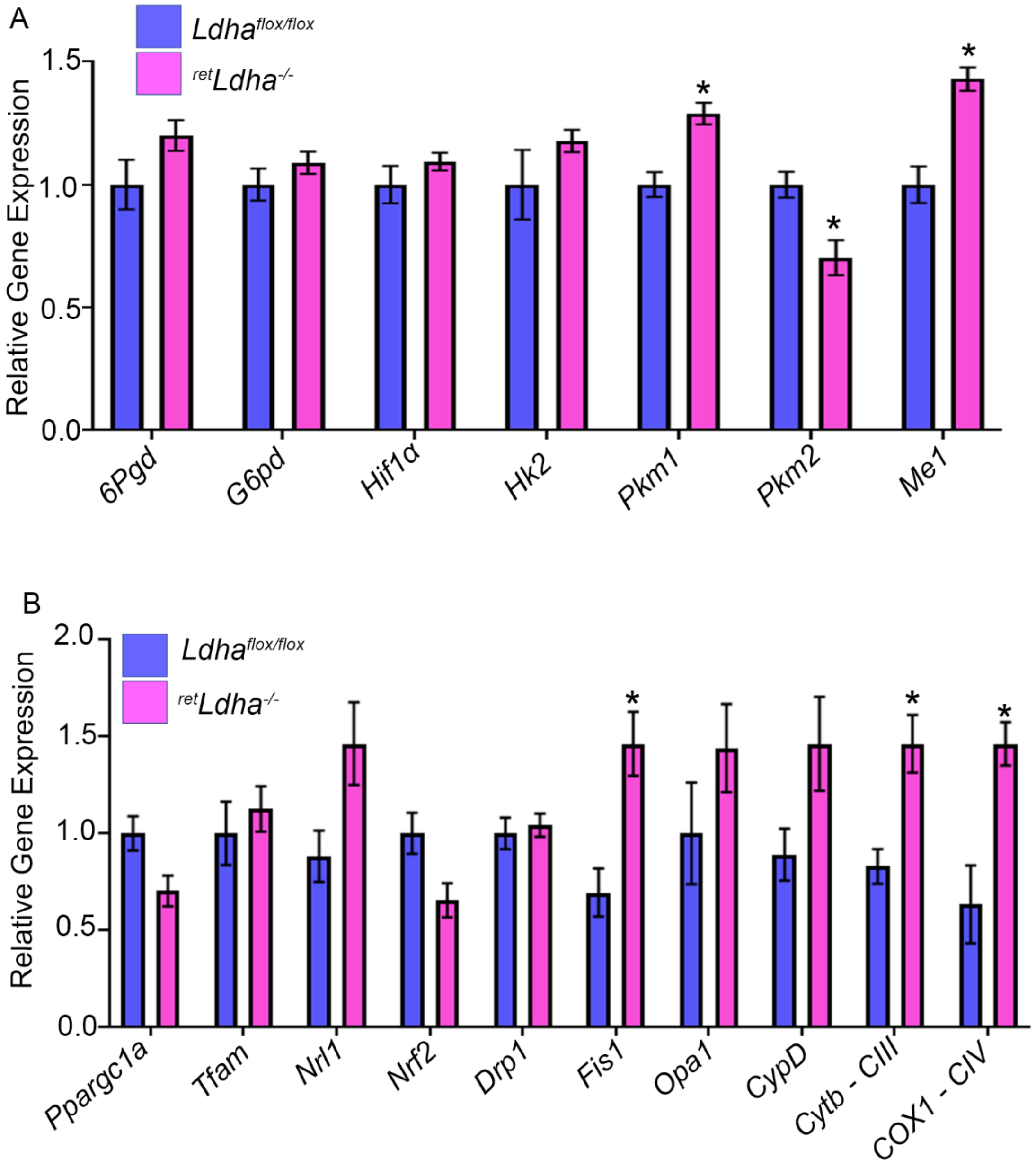

Figure S4. Quantitative real-time PCR analysis to examine the expression of genes related to mitochondrial biogenesis, mitochondrial dynamics, mitochondrial-encoded electron transport chain glycolysis, and the pentose phosphate pathway. Equal amounts of retinal mRNA from three independent 12-week-old *Ldha*$^{flox/flox}$ and ^{ret}*Ldha*$^{-/-}$ mice were used for real-time (RT)-PCR and normalized by β-actin levels (glycolysis and pentose phosphate pathway genes, **A;** mitochondrial genes, **B**). The data are mean ± *SEM* (*n*=3). *p<0.05. *Ppargc1a*, peroxisome proliferator-activated receptor gamma coactivator 1-alpha; *Tfam*, mitochondrial transcription factor A; *Nrf*, nuclear respiratory factor; *Drp1*, dynamic-related protein 1; *Fis1*, fission 1; *Opa1*, Optic atrophy protein 1; *CypD*, peptidylprolyl isomerase D; *Cytb*, mitochondria-encoded cytochrome B; *COX1*, mitochondria-encoded cytochrome c oxidase; *Actb*, beta-actin; *6pgd,* 6-phosphogluconate dehydrogenase; *G6pd*, glucose 6-phosphate dehydrogenase; *Hif1α*, Hypoxia-inducible factor 1-alpha, also known as HIF-1-alpha; *Hk2*, hexokinase 2; *Pkm1*, pyruvate kinase M1 isoform; *Pkm2*, pyruvate kinase isoform M2; *Me1*, NADP-dependent malic enzyme encoded by the ME1 gene.

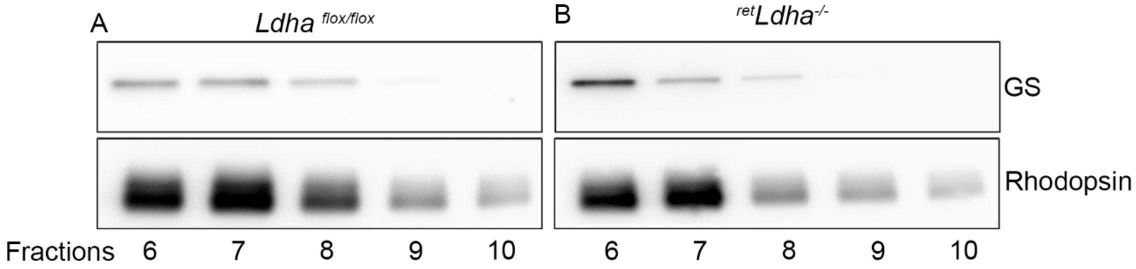

Figure S5. Glutamine synthetase expression in rods. Retinal homogenates from *Ldha*$^{flox/flox}$ (**A**) and ^{ret}*Ldha*$^{-/-}$ **(B)** mouse retinas were subjected to OptiPrep™ (8-40%) density gradient centrifugation. Fractions of intact photoreceptors were collected and subjected to immunoblot analysis with glutamine synthetase (GS) and rhodopsin.

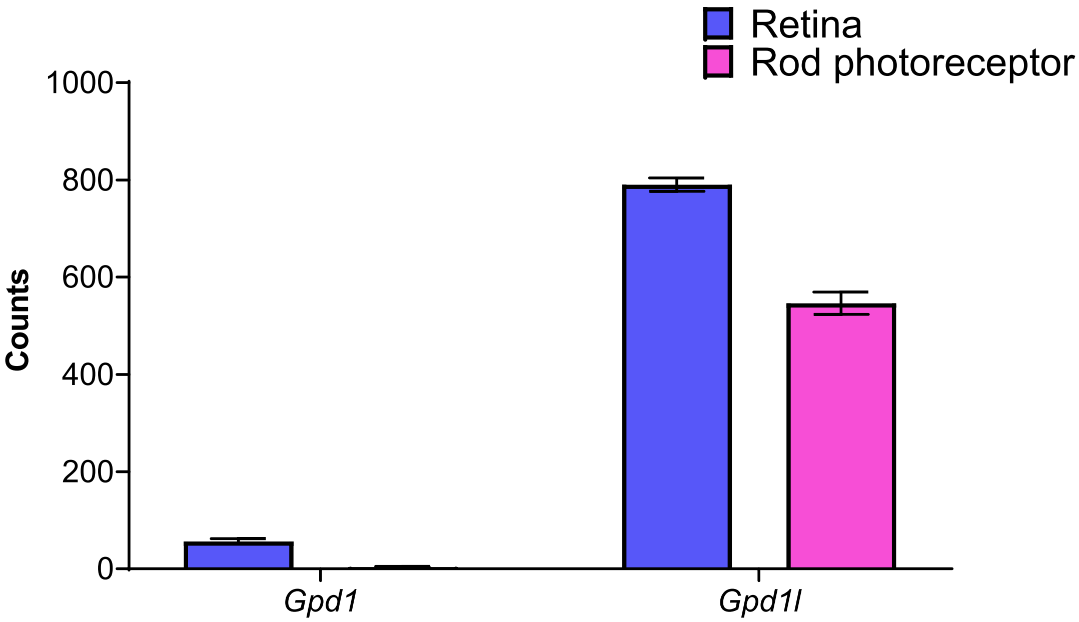

Figure S6. Expression of *Gpd1*, and *Gpd1l* in rod photoreceptor cells. The RiboTag mice carrying HA epitope-tagged ribosomal protein (*Rpl22*^{HA}) were bred with rod-specific rhodopsin-Cre and carried out the affinity purification of HA-tagged ribosomes to isolate actively translating mRNAs from rod photoreceptor cells followed by RNA sequencing. Data are mean $\pm$ *SEM (n=3)*.

Table S1 Steady-state level metabolities

GC-MS data normalized to an internal standard (ribitol) and sample weight.

Name	WT1	WT2	WT3	WT4	KO1	KO2	KO3	KO4
Pyruvate	0.002812	0.001658	0.001795	0.001811	0.002497	0.002813	0.002387	0.002928
Lactate	0.261007	0.168349	0.31737	0.248909	0.112613	0.190718	0.148341	0.146419
Ala	0.020444	0.013119	0.013377	0.018972	0.019544	0.017714	0.015242	0.019877
3Hydroxyb	0.000804	0.001226	0.001549	0.001193	0.001123	0.00123	0.001372	0.000996
Ethanimidi	0.054191	0.046996	0.04557	0.07508	0.039264	0.043779	0.043395	0.053347
NA_01	0.006029	0.004794	0.004997	0.007301	0.005061	0.004609	0.004683	0.006996
Val	0.006588	0.004775	0.003978	0.007751	0.005166	0.007335	0.003503	0.005603
Urea	0.082064	0.075945	0.090104	0.093496	0.074088	0.08092	0.067262	0.095593
Leu	0.006012	0.004022	0.004355	0.007952	0.004987	0.006892	0.003229	0.004213
Pro	0.006268	0.005376	0.00462	0.008776	0.003923	0.004091	0.003599	0.004213
Gly	0.045031	0.028362	0.031518	0.032397	0.027203	0.02335	0.032293	0.036931
Succinate	0.013116	0.007149	0.010988	0.010418	0.006255	0.009821	0.007054	0.008295
Fumarate	0.002735	0.001543	0.001524	0.001574	0.001905	0.002299	0.001827	0.002522
Toluic_acid	0.002913	0.002606	0.002766	0.003758	0.003541	0.002877	0.003265	0.00395
Uracil	0.00222	0.002069	0.001761	0.002831	0.002506	0.003665	0.001695	0.001828
Ser	0.006095	0.003376	0.003728	0.006084	0.006907	0.005973	0.004877	0.006226
NA_03	0.008006	0.004581	0.00489	0.004417	0.004666	0.005257	0.004056	0.005565
NA_04	0.001649	0.001156	0.001096	0.00109	0.001438	0.001386	0.00107	0.001336
Thr	0.002556	0.001164	0.001259	0.002046	0.002626	0.001973	0.001756	0.001874
Carbamic_a	0.001386	0.001177	0.001322	0.001524	0.001543	0.001137	0.001332	0.001608
Catechol	0.02724	0.027757	0.023468	0.041821	0.029295	0.031231	0.019306	0.037349

Cyclamic_a	0.001793	0.002739	0.002642	0.003786	0.004571	0.002725	0.002809	0.004514
Aminomalc	0.004316	0.002738	0.002729	0.002665	0.003015	0.002035	0.002825	0.00313
Malate	0.004315	0.003405	0.003255	0.003142	0.004195	0.004489	0.004153	0.00645
Oxoproline	0.111877	0.090032	0.086443	0.112363	0.097233	0.092731	0.088563	0.133624
Asp	0.058348	0.054682	0.055809	0.08312	0.05289	0.058665	0.053864	0.087228
GABA	0.077984	0.066861	0.077697	0.107931	0.058934	0.061777	0.069744	0.096242
Creatinine	0.007427	0.008132	0.008026	0.012102	0.007181	0.008666	0.007342	0.011495
Threonate	0.000964	0.001187	0.000908	0.001072	0.001375	0.001225	0.001538	0.001883
Gln	0.000444	0.000531	0.000276	0.000524	0.000751	0.000712	0.000402	0.000658
Glu	0.182404	0.162231	0.138831	0.220451	0.167203	0.185759	0.163091	0.240962
NA_05	0.009162	0.008911	0.009242	0.012335	0.008466	0.008567	0.008656	0.013418
Taurine	0.987172	1.142993	1.03849	1.72792	0.921551	1.141722	0.999415	1.550851
2Aminoadi	0.001799	0.00117	0.001481	0.00133	0.001001	0.001001	0.001074	0.001872
Amoxicillin	0.003398	0.002042	0.002307	0.003566	0.003086	0.001988	0.00179	0.002288
Phosphoric	0.030433	0.022416	0.020208	0.03545	0.03024	0.034356	0.024868	0.036319
Citrate	0.010056	0.012001	0.005694	0.007056	0.010623	0.009873	0.010293	0.016579
Lys	0.006239	0.00363	0.003442	0.005202	0.005062	0.004415	0.004141	0.004569
Fructose	0.001265	0.000839	0.000652	0.000514	0.001701	0.001743	0.001463	0.002112
Glucose_01	0.150456	0.179918	0.095214	0.141863	0.214373	0.197624	0.219938	0.324242
Ascorbate	0.012526	0.012436	0.012921	0.018072	0.008614	0.013481	0.008854	0.012338
Palmitate	0.028434	0.024347	0.025084	0.037225	0.031295	0.026829	0.025773	0.032506
Myo_Inosit	0.186291	0.148708	0.125625	0.20984	0.141714	0.174192	0.122871	0.170146
Oleate	0.00318	0.001593	0.001478	0.002366	0.002025	0.003019	0.001516	0.001449
Stearate	0.018281	0.019092	0.020047	0.030549	0.023846	0.021934	0.019696	0.02413

Fructose_6	0.000376	0.000515	0.000392	0.000222	0.000636	0.000579	0.000494	0.000697
Glucose_6I	0.000991	0.000928	0.001202	0.000842	0.001508	0.001559	0.001072	0.001464
Ile	0.003432	0.00227	0.002166	0.00415	0.002651	0.003328	0.001827	0.002704

Table S2: Interaction analysis between metabolites

ID	Name	Degree	Betweeness
C00002	Adenosine triphosphate	8	8.16
C00025	L-Glutamate	6	2.5
C00085	Fructose 6-phosphate	6	2
C00092	Glucose 6-phosphate	5	0.66
C00064	L-Glutamine	4	0.33
C00148	L-Proline	4	0.33
C00221	D-Glucose	4	0
C02336	D-Fructose	4	0
C00186	L-Lactic acid	3	0

Table S3: Real-time PCR primers for genes involved in glycolysis and the pentose phosphate pathway

Gene	Forward Primer	Reverse Primer
6Pgd	AGACAGGCAGCCACTGAGTT	AAGTTCTGGGTTTCGCTCAA
G6pd	CCTACCATCTGGTGGCTGTT	TGGCTTTAAAGAAGGGCTCA
Hif1α	GATGACGGCGACATGGTTTAC	CTCACTGGGCCATTTCTGTGT
Hk-2	GGAACCCAGCTGTTTGACCA	CAGGGGAACGAGAAGGTGAAA
Pkm1	GCCTCCAGTCACTCCACAGA	CAGCACGGCATCCTTACACA
Pkm2	CAGCACCTGATTGCCCGAGA	CCAGACTTGGTGAGCACGATA
Me1	AGAGGTGTTTGCCCATGAAC	GCTGGTCGGATTACTCAAAGC
Actb	ACTGGGACGACATGGAGAAG	GGGGTGTTGAAGGTCTCAAA

Abbreviations: *6pgd,* 6-phosphogluconate dehydrogenase; *G6pd*, glucose 6-phosphate dehydrogenase; *Hif1α*, Hypoxia-inducible factor 1-alpha, also known as HIF-1-alpha; *Hk2*, hexokinase 2; *Pkm1*, pyruvate kinase M1 isoform; *Pkm2*, pyruvate kinase isoform M2; *Me1*, NADP-dependent malic enzyme encoded by the ME1 gene; *Actb,* beta-actin.

Table S4: Real-Time PCR primers for mitochondrial biogenesis, mitochondrial dynamics and mitochondrial-encoded electron transport chain genes

Gene	Forward primer	Reverse primer
Mitochondrial biogenesis		
Ppargc1a	GCAGTCGCAACATGCTCAAG	GGGAACCCTTGGGGTCATTT
Tfam	TCCACAGAACAGCTACCCAA	CCACAGGGCTGCAATTTTCC
Nrf1	AGAAACGGAAACGGCCTCAT	CATCCAACGTGGCTCTGAGT
Nrf2	ATGGAGCAAGTTTGGCAGGA	GCTGGGAACAGCGGTAGTAT
Mitochondrial dynamics		
Drp1	ATGCCAGCAAGTCCACAGAA	TGTTCTCGGGCAGACAGTTT
Fis1	CAAAGAGGAACAGCGGGACT	CAAAGAGGAACAGCGGGACT
Opa1	ACCTTGCCAGTTTAGCTCCC	TTGGGACCTGCAGTGAAGAA
CypD	AGATGTCAAATTGGCAGGGGG	TGCGCTTTTCGGTATAGTGCT
Mitochondrial-encoded electron transports chain genes		
Cytb-CIII	GGCTACGTCCTTCCATGAGG	TGGGATGGCTGATAGGAGGT
COX1-CIV	ATCACTACCAGTGCTAGCCG	CCTCCAGCGGGATCAAAGAA
Actb	AGAAGCTGTGCTATGTTGCTCTA	TCAGGCAGCTCATAGCTCTTC

Abbreviations: *Ppargc1a*, peroxisome proliferator-activated receptor gamma coactivator 1-alpha; *Tfam*, mitochondrial transcription factor A; *Nrf,* nuclear respiratory factor; *Drp1*, dynamic-related protein 1; *Fis1,* fission 1; *Opa1*, Optic atrophy protein 1; *CypD*, peptidylprolyl isomerase D; *Cytb*, mitochondria-encoded cytochrome B; *COX1*, mitochondrial-encoded cytochrome c oxidase, *Actb,* beta actin.

Table S5. Antibodies used for immunofluorescence (IF) and immunoblot Analysis

Antibody raised against	Host species	Dilution	Manufacturer	Catalog number
LDHA	Rabbit	1:1000<br>1:50 (IF)	Proteintech	21799-1-AP
LDHB	Rabbit	1:1000<br>1:50 (IF)	Proteintech	14824-1-AP
Pde6β	Mouse	1:1000<br>1:25 (IF)	Santa Cruz	SC-377486
Rhodopsin	Mouse	1:1000<br>1:50 (IF)	In-house	Gift from Dr. Jim McGinnis (OUHSC)
Rod-Arrestin	Mouse	1:1000<br>1:500 (IF)	In-house	Gift from Dr. Paul Hargrave (University of Florida)
M-opsin	Rabbit	1:1000<br>1:100 (IF)	Millipore Sigma	AB5405
S-opsin	Rabbit	1: 100 (IF)	Millipore Sigma	ABN1660-1
Cone-Arrestin	Rabbit	1:1000	Millipore Sigma	AB15282
Actin	Mouse	1:1000 (IB)	Thermo Fisher Scientific	MA1-744
Glutamine synthetase (GS)	Mouse	1:1000<br>1:50 (IF)	Abcam	Ab64613
Glial fibrillary acid protein (GFAP)	Rabbit	1:100 (IF)	Dako	20334
PKM1	Rabbit mAb	1:1000<br>1:50 (IF)	Cell Signaling Technology	7067
PKM2	Rabbit mAb	1:1000<br>1:100 (IF)	Cell Signaling Technology	4053
Phospho-PKM2 (Tyr105)	Rabbit	1:1000<br>1:50 (IF)	Cell Signaling Technology	3827
Hexokinase 1	Rabbit mAb	1:1000<br>1:25 (IF)	Cell Signaling Technology	2024
Hexokinase II	Rabbit mAb	1:1000<br>1:25 (IF)	Cell Signaling Technology	2867
PDH	Rabbit mAb	1:1000	Cell Signaling Technology	3205
Aldolase C	Mouse	1:1000<br>1:100 (IF)	EnCor Biotechnology, Inc.	MCA-4A9
Glut1	Rabbit	1:1000<br>1:25 (IF)	Novus Biologicals	NB110-39113

Table S6: Real-time PCR primers to quantitate the expression of *Ldha* and *Ldhb* in rod photoreceptor, Müller, and RPE cells

Gene	Forward Primer	Reverse Primer
Ldha	ACTGCAGGCTTCGATTACCC	ATGGACGTACACACTGGAGC
Ldhab	GGATTCACCCCGTGTCTACC	GAGCGACCTCATCGTCCTTC
Rpl38	CGCCATGCCTCGGAAA	CCGCCGGGCTGTCAG
Actb (*β-actin*)	ACTGGGACGACATGGAGAAG	GGGGTGTTGAAGGTCTCAAA
